# Spatially Programmable Chirality in Cellulose Nanocrystal Films via Rotational Magnetic Flow

**DOI:** 10.1021/acsami.5c12750

**Published:** 2025-08-30

**Authors:** Jisoo Jeon, Dhriti Nepal, Michael E. McConney, Timothy J. Bunning, Vladimir V. Tsukruk

**Affiliations:** 1 School of Materials Science and Engineering, 1372 Georgia Institute of Technology, Atlanta, Georgia 30332, United States; 2 Air Force Research Laboratory, 33319 Wright-Patterson Air Force Base, Ohio 45433, United States

**Keywords:** magnetic nanoparticles, cellulose nanocrystals, programming alignments, dynamic stimuli, programmed chiroptical appearance

## Abstract

Programmed assembly of natural materials on a large scale is often limited by inherent factors, including dimensional dispersity, complex hierarchical organization, and slow processing kinetics. In this study, we demonstrate a scalable strategy to preprogram the chiral assembly of cellulose nanocrystals (CNCs) by applying a rotational magnetic field during evaporation-induced self-assembly. To facilitate magnetic responsiveness, CNCs are decorated with magnetic nanoparticles and subjected to a rotational magnetic field. This magnetically induced azimuthal shear flow aligns the nanocrystals with a remarkably high local orientational order parameter of 0.96. On the macroscopic scale, the rotational flow generates a gradual, azimuthal alignment, resulting in large-area orientational ordering with identical helicity extending across centimeter-scale regions. Notably, the handedness of the chiral structure and the emergence of distinct optical textures, such as centimeter-wide Maltese crosses, can be controlled by adjusting the direction and strength of the induced large rotational magnetic vortex. This approach provides a versatile route for the larger-scale fabrication of programmable chiral photonic materials from bioderived building blocks.

## Graphical abstract

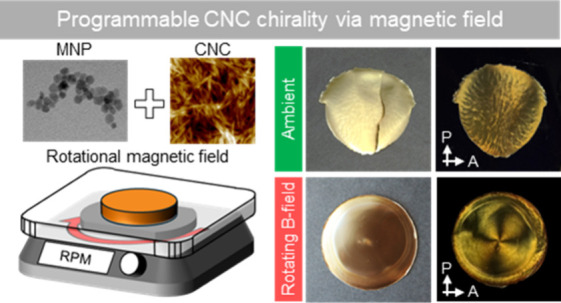

## Introduction

Anisotropic and low-dimensional nanomaterial components, such as nanosheets or needles, offer versatile pathways to control their alignment, assembly, and phase behavior through external stimuli or templating strategies. A prominent example is liquid crystal (LC) materials, which exhibit anisotropic mechanical and photonic properties after alignment.^1–3^ The alignment can be programmed using external stimuli, such as shear,^4^ magnetic fields,^5^ electric fields,^6^ and different templates.^7^ Further polymerization enables applications in soft robotics,^8^ actuators,^9^ and switchable adhesives.^10,11^ Among the various optical phenomena exhibited by LC and colloidal materials, striking effects can be observed under crossed polarizers, often originating from engineered topological defects and boundary conditions.^12,13^ Point defects typically arise in confined geometries, where multiple colloidal particles or anisotropic molecules interact. For instance, a 360° rotation of needle-like colloidal particles generates a + 1 point defect.^14^ In the case of cellulose nanocrystals (CNCs), uniform azimuthal alignment results in the emergence of a distinct characteristic Maltese cross pattern under crossed polarizers, reflecting a highly ordered local monodomain organization at the microscopic scale.

CNCs derived from natural plant sources exhibit a chiral nematic LC phase in concentrated aqueous suspensions. Upon drying or freezing, CNCs retain their chiral organization, resulting in periodic helical structures that give rise to vivid iridescent colors.^15,16^ In their native self-assembly without external stimuli, CNCs typically form left-handed helices due to their intrinsic twisted morphology and asymmetric interparticle interactions. To modulate CNC alignment beyond their natural behavior, several approaches have been explored, such as shear stress,^17,18^ additives,^19–21^ capillary confined flow,^22^ and air flow.^23^ While these methods can adjust the pitch length and overall orientation of CNC domains, they offer limited control over fine-scale structural features and chirality. In contrast, traditional alignment techniques, such as electric fields or surface templating, can provide precise control but often require closed systems. These designs include sandwich cells composed of indium tin oxide (ITO)-coated electrodes^6^ or substrate-based templates such as photoaligned layers^24,25^ or patterned micro- and nanochannels.^7^

On the other hand, magnetic fields are widely used as external stimuli to program the orientation and optical properties of functional nanomaterials. To this end, CNCs possess modest diamagnetism,^26^ achieving alignment under a strong magnetic field exceeding 1 T.^27^ To improve this ability, CNCs can be functionalized with magnetic nanoparticles (MNPs), enabling their manipulation under moderate magnetic fields.^28,29^ Magnetic additives such as iron nano/microparticles,^30,31^ neodymium boron iron (NdFeB),^32,33^ or various MNPs have been incorporated into bioderived materials^34,35^ to enhance their magnetic responsiveness. This strategy allows for control over the orientation of CNC suspensions under static magnetic fields, resulting in unidirectional alignment and localized patterning. However, such approaches often suppress the intrinsic chiral organization of CNCs or are limited in scale, typically to microscopic domains.

Herein, we investigate the use of a weak rotational magnetic field to induce shearing flow in CNC/MNPs suspensions during an evaporation-induced self-assembly process. The magnetically induced azimuthal flow generates a high shear force that facilitates large-scale directional orientation within the lyotropic CNC suspension with a helical organization. This process results in the fabrication of a large-scale monodomain structure in a dried solid film. The rotational flow aligns CNCs globally along the flow direction, creating an azimuthal alignment with a +1 defect at the center. Micro- and nanoscopic surface and cross-sectional analyses revealed structural transformation in the flow-aligned chiroptical organization, showing variable chiroptical properties, which are tunable by adjusting the magnetic moment, rotation rate, and evaporation rate. The handedness (left or right) of the azimuthal patterns, along with the appearance of characteristic chiral optical signatures, such as unusually large centimeter-wide Maltese crosses, can be induced and reversed by modifying the rotational direction and strength of the magnetic vortex during the self-assembly process.

## Results and Discussion

### Patterned Film Fabrication

The magnetic stirrer was adapted to generate rotational magnetic fields, inducing rotational flow through its rotation (Figure 1a). Magnetic field simulations revealed a uniform unidirectional magnetic field around the magnetic stirrer (Figure 1d, see Experimental Section). The magnetic flux density at the center of the magnetic stirrer was approximately 20 mT.

**Figure 1:**
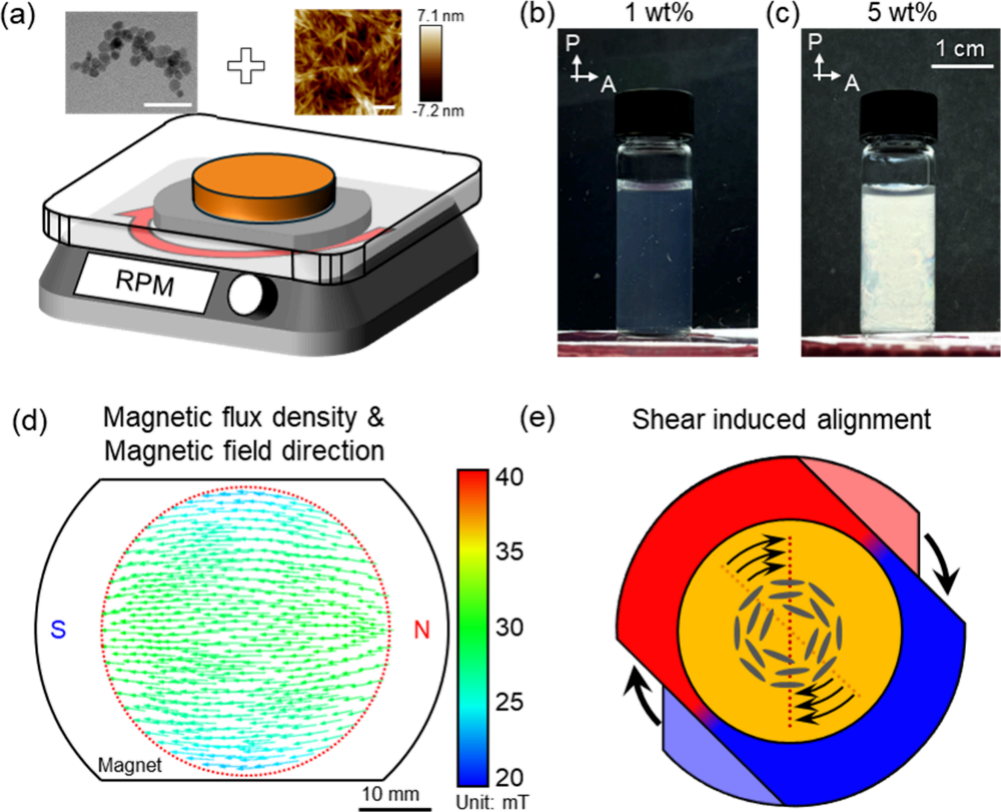
(a) TEM image of iron oxide nanoparticles, AFM topography of CNCs, and schematic illustration of the fabrication process using a magnetic stirrer. Scale bars, TEM: 40 nm, AFM: 200 nm. Crossed polarized images of CNC suspensions with (b) 1 and (c) 5 wt % CNC concentration, identical magnification. (d) Simulation of the magnetic field distribution within the magnetic stirrer. A red dot circle indicates the size of a Petri dish, which was used for film formation. (e) Schematic illustration of azimuthal orientation induced within the CNC suspension by the rotational magnetic field, as discussed below.

The synthesized MNPs had an average diameter of 7.0 ± 2.3 nm that represents the dimensions needed for nanocrystal decoration with the ability to be aligned in low magnetic fields, as was shown in our prior publications.^36^ CNCs were extracted from wood pulp through hydrolysis processing established in our laboratory (see Experimental section).^37^ The CNC suspension and iron oxide nanoparticle suspension were mixed until the total concentration of CNC/MNP in suspension reached 5 wt %, which is within the known range (3 to 7 wt %) to have lyotropic LC phases (Figure 1b,c).^38,39^ The concentrated LC suspension was then poured into a Petri dish (35 mm in diameter), placed in the center of a magnetic stirrer, and allowed to undergo an evaporation-induced self-assembly process under a continuously rotating magnetic field (Figure 1e). After complete drying, the films were collected, and the freely standing films were further characterized.

### Morphology, Orientation, and Optical Appearance

The conventional CNC film and CNC/MNP composite film formed under ambient conditions show a common optical appearance in unpolarized light and in crossed polarized images by random Brownian motion (Figure 2 and Figure S1).^40^ In static and uniform magnetic fields, the MNPS are aligned along the magnetic field, forming brown stripes, which form a CNC/MNP composite film with a unidirectional nematic alignment (Figure 2 and Figure S2). Under crossed polarizers, the 45° directed film is brighter than the other, which indicates the film has unidirectional alignment (Figure S2b). However, the brightness change is not uniform, indicating that the film has low nematic order. At the microscopic scale, the patterns are also unidirectional with birefringent color, which means the chiral colloid of CNCs is arranged along the MNP stripe pattern (Figures S2c, S3, and S4).

**Figure 2:**
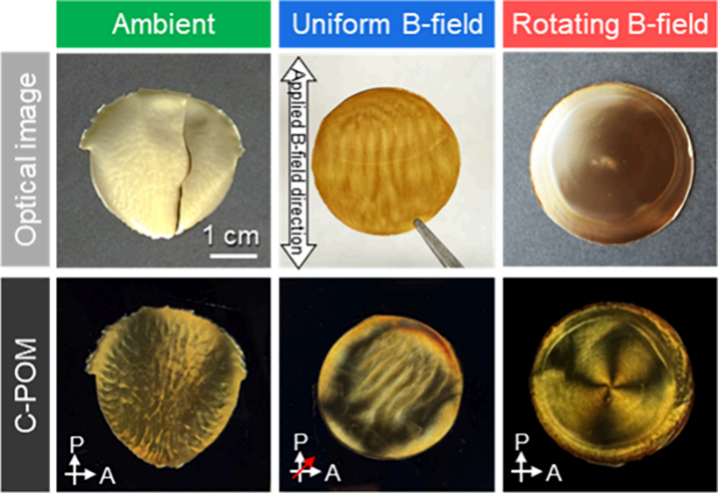
Unpolarized optical images and crossed polarized images of CNC/MNP composite film evaporated (left) without, (center) static uniform, and (right) rotational magnetic field. Concentration of MNPs in composite films is 2.0 wt %. All of the optical images share the same scale bar. Red arrow indicates the direction of the applied magnetic field.

Since the surface of CNCs is modified with citric acid, the hydrogen bonding between CNCs and MNPs induces the arrangement of CNC colloids. Apparent brightness differences with different angles between two crossed polarizers indicate that CNCs have a unidirectional arrangement. Clear angle-dependent light scattering was observed in the incident angle, which also proves that the film has a unidirectional CNC arrangement (Figures S2e,f). When a rotational magnetic field is applied during evaporation, the resulting film displays a distinct circular optical pattern and a characteristic azimuthal pattern in the crossed polarized image, known as the Maltese cross (Figure 2).^41^ In the case of a larger diameter Petri dish, a brown ring composed of MNPs is formed at the area with the edge of the magnet, which has a higher magnetic flux density, ∼30 mT, than the center area, 20 mT (Figure S5). This allows the MNPS to move areas with a higher magnetic flux density, forming a less clear pattern. These signature optical patterns arise from the preferential azimuthal orientation of CNCs, as discussed below.

The regular CNC/MNP composite film obtained without magnetic field rotation does not exhibit unidirectional flow patterns in optical microscopy (OM) and periodic brightness changes along with the whole composite film in crossed polarized optical microscopy (C-POM) (Figure 3 and Figure S6). In contrast, clear flow patterns oriented in the direction of rotation are observed in the CNC/MNP composite film obtained under a rotational magnetic field. Additionally, varying brightness at specific angles, such as (2*n* – 1) × π/4, indicates bright colors, suggesting that the CNCs are oriented at the ±45° azimuthal angle relative to the polarizer/analyzer. Angles of 2­(n – 1) × π/4 show dark colors, indicating parallel alignment of CNCs with the polarizer/analyzer orientation (Figure 3b and Figure S7).

**Figure 3:**
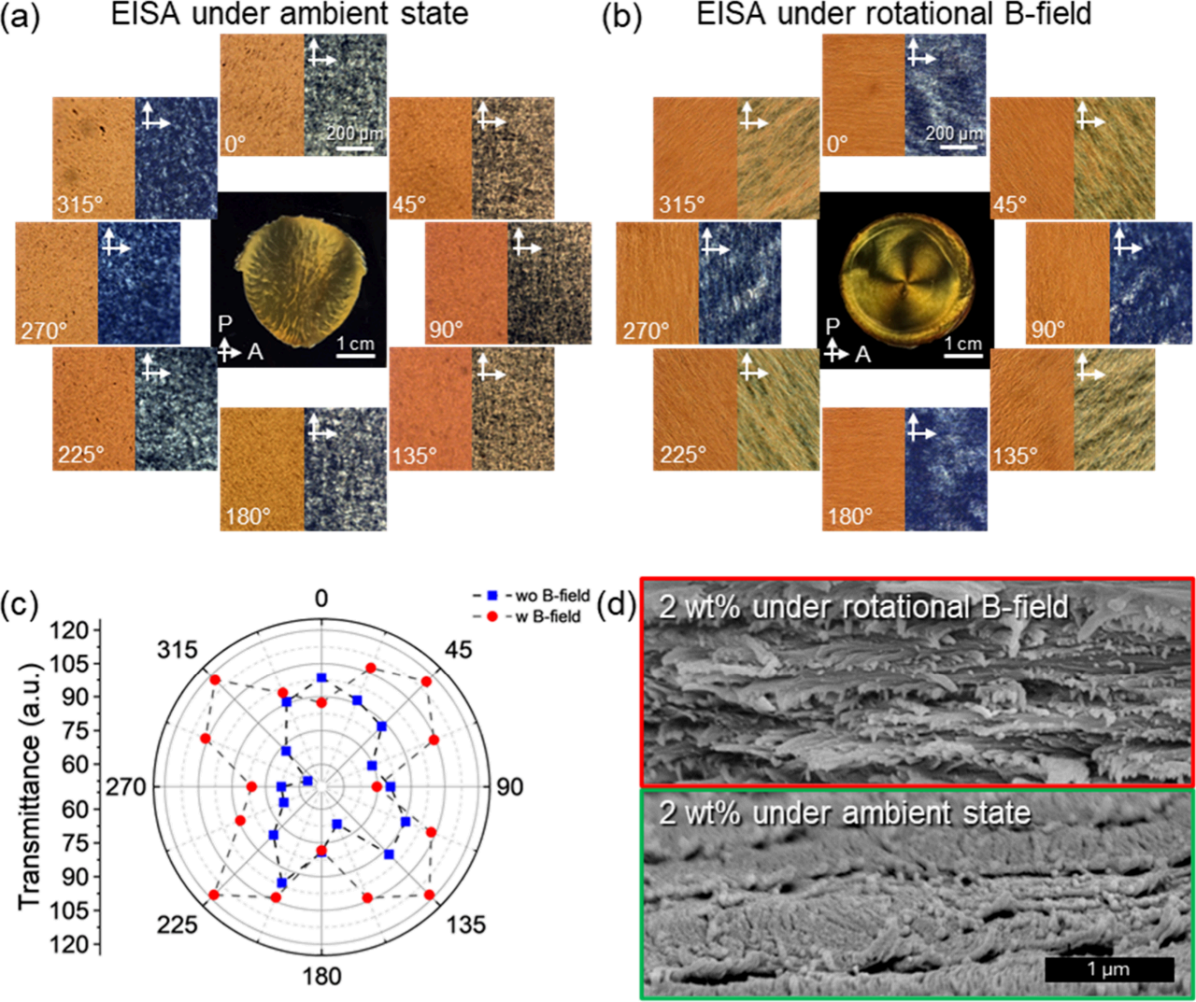
(Left) Optical and (right) crossed polarized optical micrographs of CNC/MNP composite evaporated under ambient (a) states and (b) rotational magnetic field with (center) crossed optical images. The applied rpm was 200 rpm. All the optical micrographs share the scale bar. (c) Transmittance of light through the CNC/MNP composite films between crossed polarizers. (d) Cross-sectional SEM images of 2 wt % CNC/MNP composite film evaporated under a rotational magnetic field (top) and without a magnetic field (bottom). Magnification is identical for both images.

The composite films evaporated under a rotational magnetic field show a distinct four-petal flower pattern (known as a Maltese cross), as summarized in the light transmittance diagram in Figure 3c. To further confirm the alignment of CNCs, the cross-section of the film was examined using scanning electron microscopy (SEM) (Figure 3d). The SEM images reveal a unidirectional arrangement of CNCs along the flow direction, displaying a highly oriented characteristic Bouligand morphology that is different from that of regular CNC films (Figure 3d).

The surface topographies of CNC/MNP films were also analyzed at different angular positions (Figure 4 and Figures S8 and S9). To quantify the alignment of CNCs, orientational ordering was analyzed using GTfiber image analysis.^42^ The direction of CNC alignment as measured by AFM image analysis corresponds to the flow directions at these positions that are expected to align with the 0/180 and 90/270° angles. At the 45/225 and 135/315° angles, CNCs are oriented diagonally, matching the tangent direction of the rotational flow, as well.

**Figure 4:**
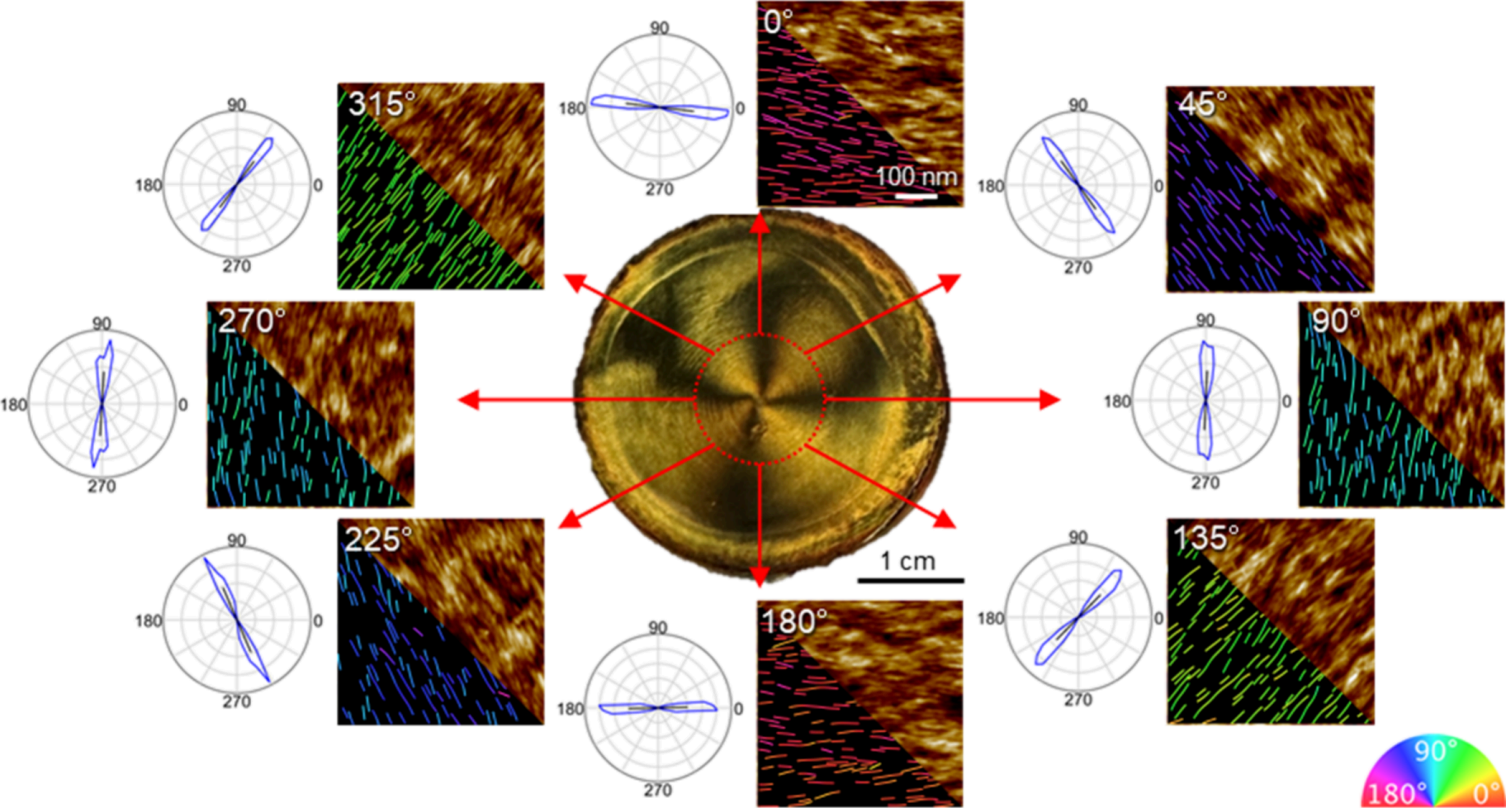
AFM topography with color-coded orientation analysis for each position of CNC/MNP composite film evaporated under a rotational magnetic field at 200 rpm. Inset graphs are azimuthal distributions of orientational order obtained from image analysis. The scale bar on the top AFM image of 100 nm holds for all AFM images. The azimuthal distributions and polar plots show color-coded directionalities (right bottom inset image).

Notably, the local orientational order parameter reaches 0.96 ± 0.02, approaching the theoretical limit of 1.0,^43^ indicating near-perfect local alignment of nanocrystals along the rotational axis. This level of ordering is significantly higher than that typically observed in conventional unoriented CNC films, which exhibit order parameters in the range of 0.7–0.8 (Figure S10).^44^

To investigate the parameters influencing the azimuthal organization, MNP concentrations varied from 0.5 to 5.0 wt % (Figures 5 and 6). In the absence of a magnetic field, films across all MNP concentrations displayed no distinct patterns under optical microscopy, instead exhibiting typical fingerprint textures and iridescent colors characteristic of CNC assemblies (Figure S11).

**Figure 5:**
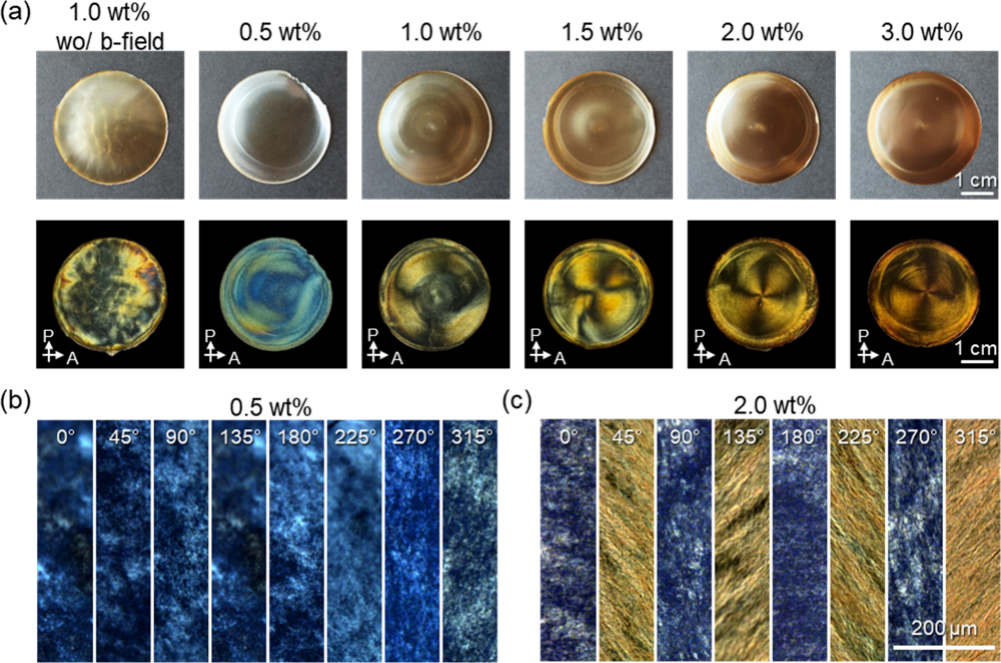
(a) Unpolarized optical (top) and crossed polarized (bottom) optical images according to the concentration of MNPs in the composite films. Each sample is indicated by the top name. Variation of MNP concentration in composite films: 0.5, 1.0, 1.5, 2.0, and 3.0 wt %, respectively. All the images share the same scale bar. C-POM images with (b) 0.5 wt % and (c) 2.0 wt % composite films. Scale bar in (c) is the same for (b).

**Figure 6:**
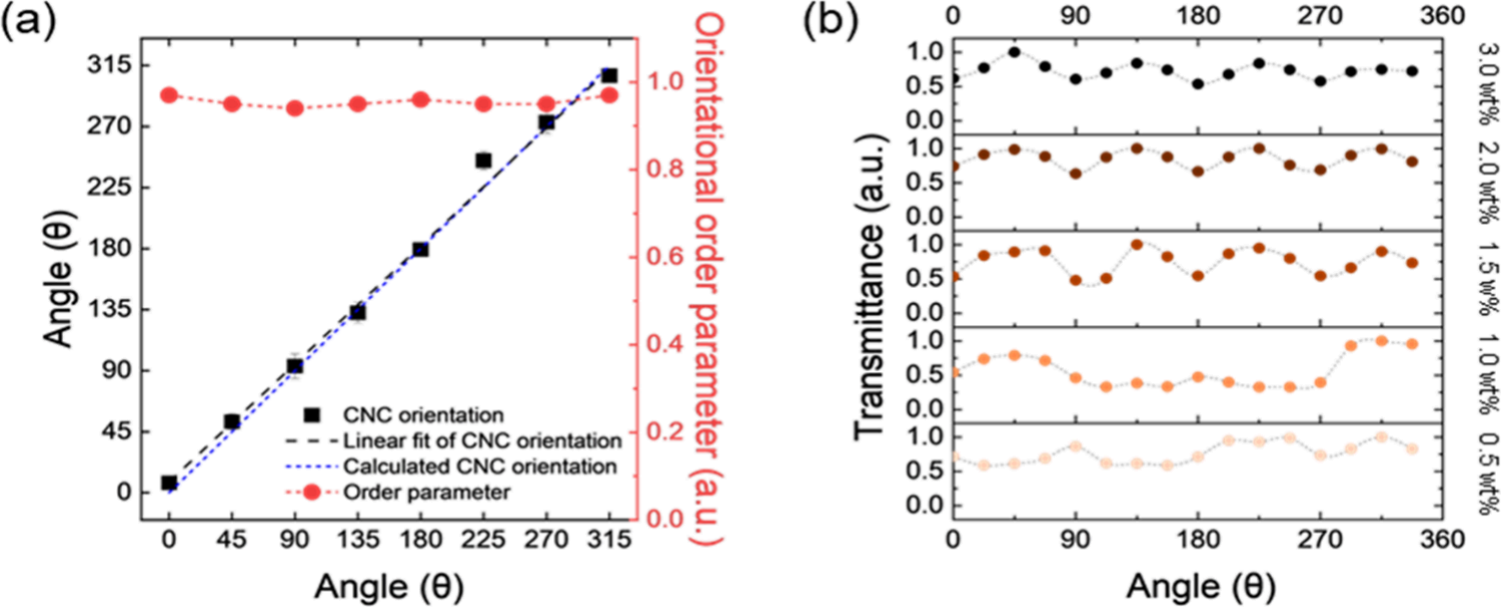
(a) Measured and calculated CNC aligned angles and orientational order parameters depending on angular positions. (b) Angular variation of transmittance from crossed polarized optical micrographs for different MNP loadings.

In contrast, CNC/MNP films formed under a rotating magnetic field exhibit distinctive centimeter-scale azimuthal patterns at MNP loadings of 2.0 and 3.0 wt %, as evidenced by a clear crossed Maltese cross pattern in C-POM images. These optical features indicate a uniform concentric alignment of CNCs across the entire film. At a higher MNP concentration (5.0 wt %), particle aggregation disrupts this organization, resulting in loss of the defined optical pattern (Figure S12). At lower MNP loading (<1.5 wt %), the films predominantly exhibit bluish iridescent color and the characteristic Bouligand morphology typical of helical organization (Figures 5a, S13 and S14). Notably, composite films with 2.0 and 3.0 wt % MNP display unidirectionally aligned CNC morphologies and a preferential birefringence orientation, particularly evident at these intermediate concentrations (Figure 5b,c and Figures S15–S20). Clear azimuthal patterns were observed in different evaporation rates from different humidities adapted, which confirms reliable pattern formation (Figure S21).

The orientational order derived from AFM image analysis remains consistently high across all azimuthal positions with values of 0.96 ± 0.02. Moreover, the variation of local orientation angle closely follows the expected azimuthal direction, deviating by no more than ± 7° (Figure 6a). This strongly confirms the near-perfect azimuthal patterning of nanocrystal alignment, driven by the rotational flow.

The periodic modulation of light transmittance under crossed polarizers confirms that Maltese cross optical texture is consistently observed for magnetic nanoparticle loading from 1.5 to 3.0 wt % (Figure 6b). Finally, it is important to note that the presence of citric acid ligands on the MNPs is critical for their dispersion; omission of these ligands leads to significant aggregation and the loss of uniform azimuthal patterning (Figures S22 and S23).^45^ Citric acid on the MNP surfaces provides hydrogen bonding between the carboxyl group in citric acid and the hydroxyl group on the CNCs and water molecules, which leads to a stable dispersion state under aqueous environments.^46^ FT-IR spectra confirmed that the surface of MNPs was modified with citric acid, and they are not significantly affected to CNCs (Figure S24). Citric acid coating on the MNP surface provides carboxylate stretching near 1615 and 1405 cm^–1^, and the peaks around 3500 to 3000 cm^–1^ are from the hydroxyl group in FT-IR spectroscopy (Figure S24a).^47^ CNCs and CNC/MNP composites have no significant peak differences with or without MNPs (Figure S24b).

It is important to note that the shear rate plays a crucial role in determining the orientation alignment of nanocrystals.^48,49^ In general, a slightly higher shear rate increases the orientational order. However, CNC is unable to follow high rotation, resulting in insufficient shear within the suspension. Overall, the further increase of magnetic field rotational rates reduces the development of optical texture, including both the overall pattern definition and optical contrast, likely due to viscosity-induced lag in the suspension flow (Figure 7 and Figures S25 and S26).

**Figure 7:**
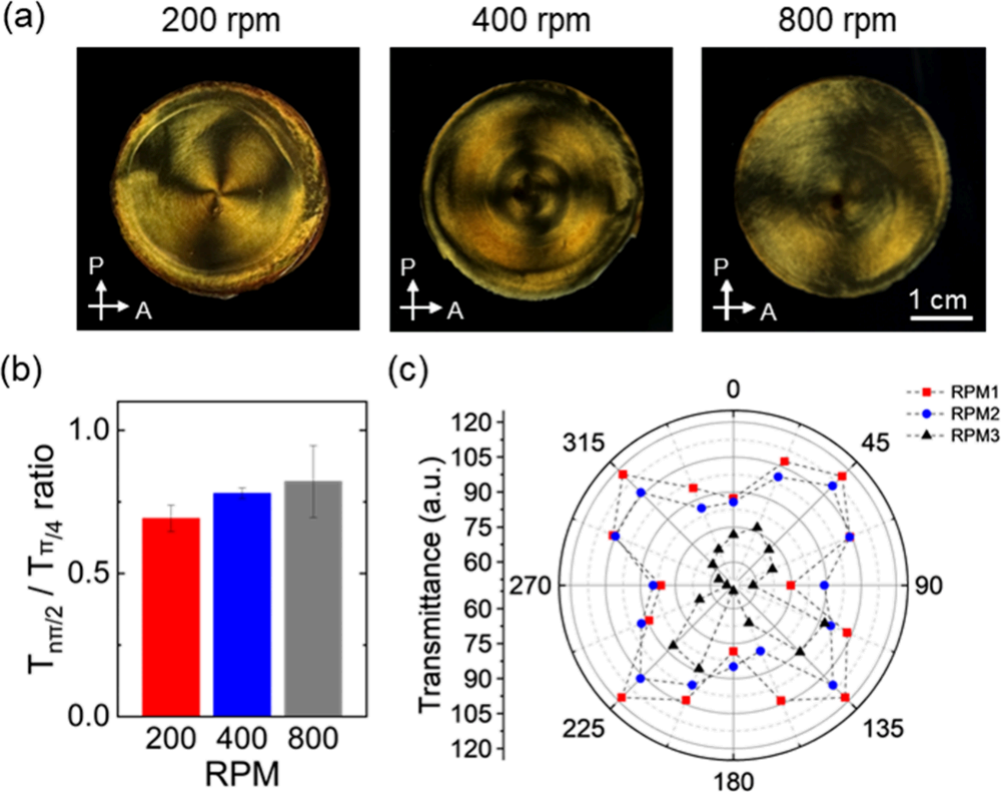
(a) Crossed polarized optical images of CNC/MNP composite films with different rpm. All of the images share the same scale bar. (b) Average ratio of light transmittance between dark areas and bright areas according to rpm. (c) Polar plot of light transmittance at different rates.

### Mechanical Performance of MNP/CNC Films

As is well-known, anisotropic CNC films exhibit direction-dependent mechanical properties, typically showing reduced strength in the transverse direction to CNC alignment.^50^ To investigate the mechanical behavior of CNC/MNP composite films formed under a rotating magnetic field, specimens were cut radially, from the edge to the center, that is, perpendicular to azimuthal CNC alignment. Stress–strain curves were obtained for these radially cut specimens (Figure 8).

**Figure 8:**
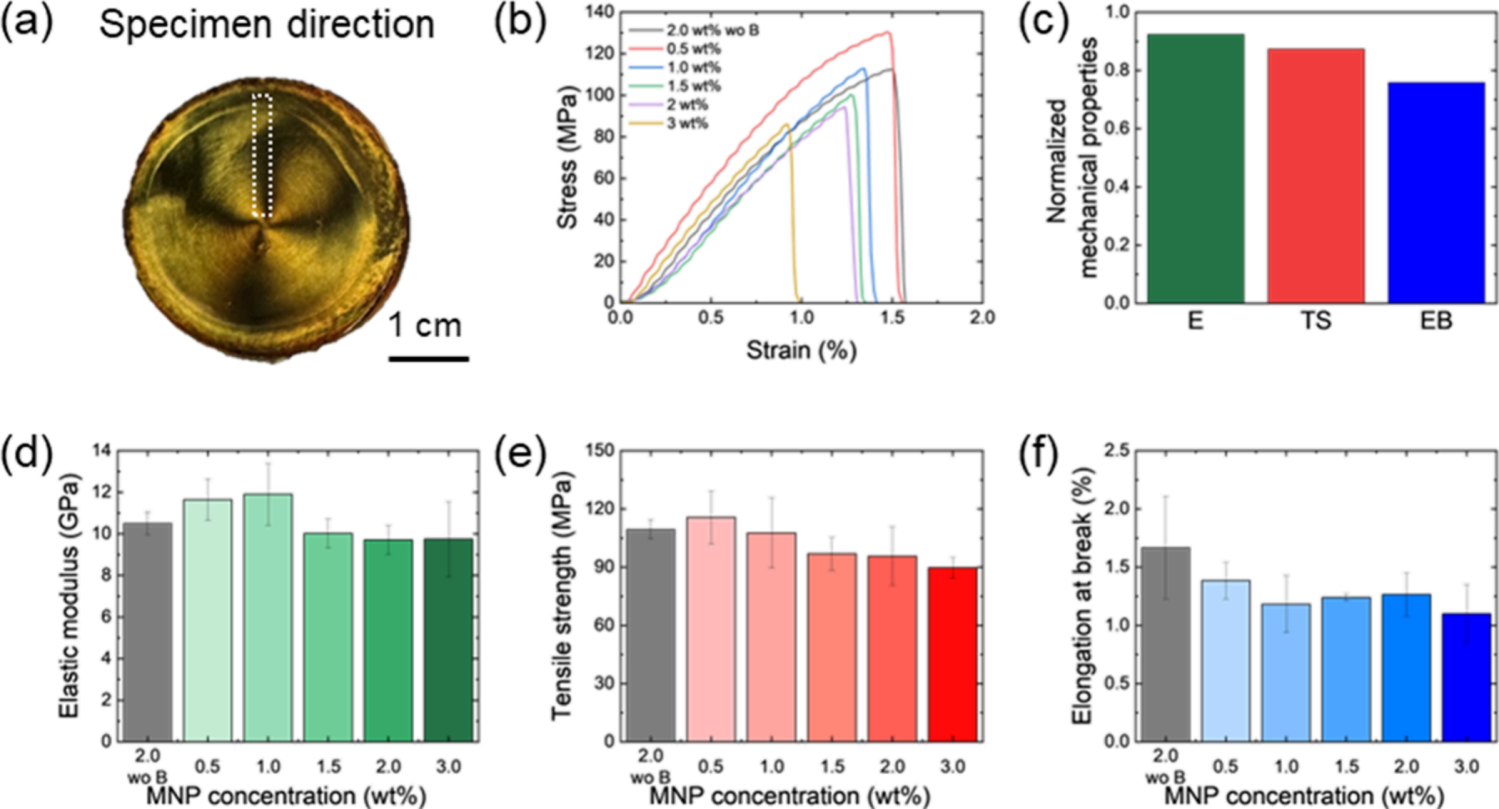
Mechanical properties of radially cut specimens from CNC/MNP composite films with different MNP loadings. (a) Position from which the specimen was taken from the film. (b) Stress–strain curves of CNC/MNP composite films. E: elastic modulus, TS: tensile strength, EB: elongation at break. (c) Normalized mechanical properties of CNC/MNP composite film evaporated under rotational magnetic field compared to those of evaporated under ambient state without magnetic field. (d) Elastic modulus, (e) tensile strength, and (f) elongation at break of radial specimens of CNC/MNP composite films.

Overall, all specimens exhibited a modest decrease in elastic moduli and toughness, by 8–15%, along with increased brittleness in comparison with traditional polydomain CNC/MNP films due to the fact that the applied stress is normal to the direction of nanocrystal alignment (Figure 8b,c).^51^ Furthermore, both the elastic modulus and mechanical strength decreased with increasing MNP concentration, likely due to nanoparticles acting as impurities that disrupt internanocrystal interactions (Figure 8d–f).^19^ As the rotation rate increases, the anisotropy of alignment in composite film decreases, which results in an increase of mechanical performance (Figure S27).

### Optical Properties and Circular Polarization

UV–Vis spectroscopy of control CNC/MNP composite films fabricated without a magnetic field revealed a progressive red shift in the main peak position, moving from 310 to 520 nm as MNP concentration increased (Figure 9a,b). At the film center, the peak shift is linear with increasing MNP content that is consistent with the well-known red-shift phenomena caused by an increase in cholesteric pitch length due to intercalated nanoparticles (Figure 9c).^52^ Notably, films with lower MNP loadings showed higher peak intensities, likely due to the presence of a more highly ordered helical structure and reduced nanoparticle-induced disorder (Figure S28). The peaks in UV–vis spectra are flattened by aggregation of MNPs, which are induced by the applied magnetic field; however, the red shift of spectra is clearly observed in the graphs.^53^

**Figure 9:**
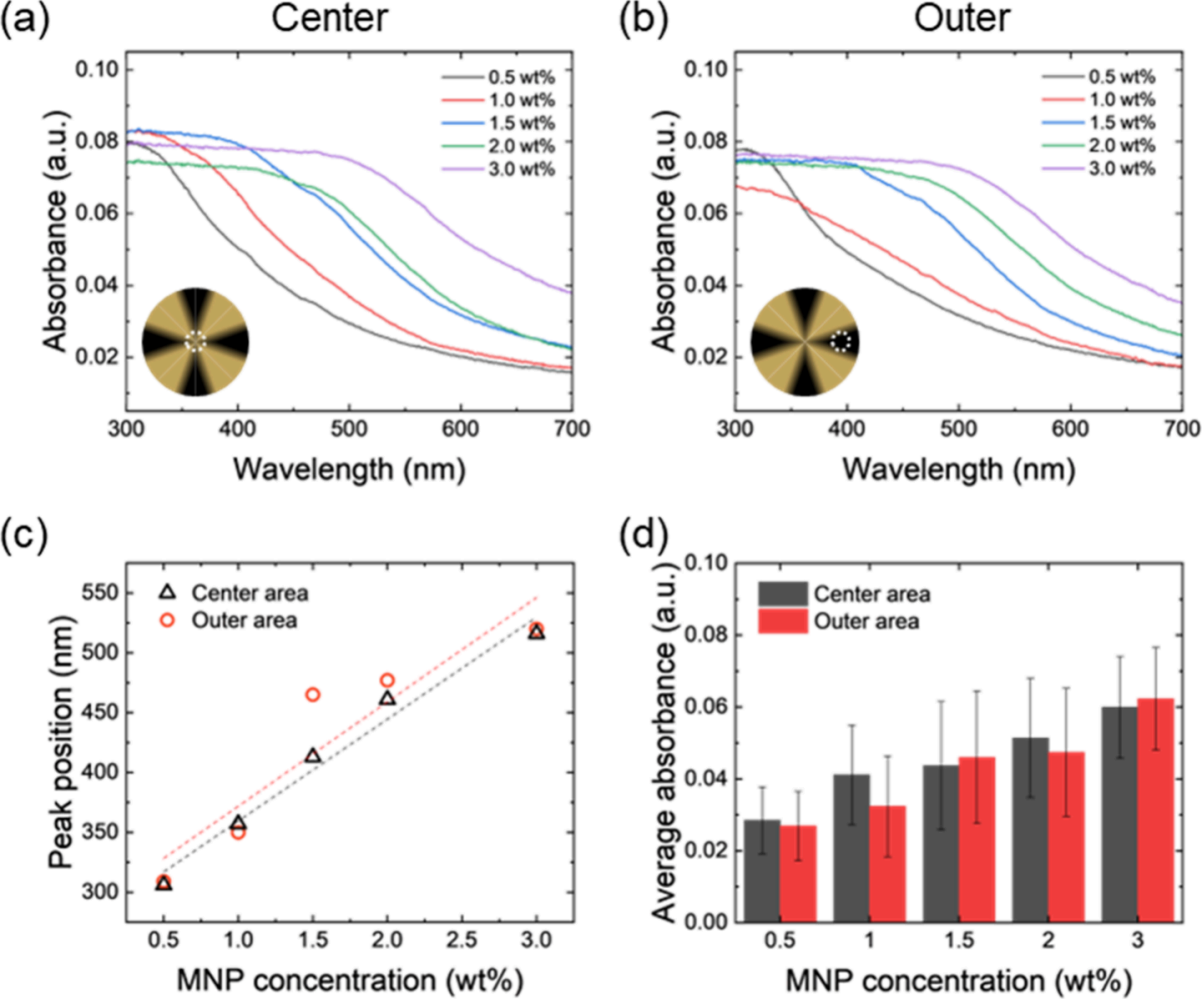
UV–Vis spectra at the (a) center and (b) outer areas of the CNC/MNP composite films with different MNP loadings. Inset schematic illustrations indicate the position of the measurement. (c) Peak positions, and (d) average absorbance in visible light (400 to 700 nm) CNC/MNP composite film evaporated under rotational magnetic field with different MNP loadings in composite film.

In contrast, CNC/MNP films fabricated under a rotational magnetic field exhibit suppressed UV–Vis peak intensities in the outer regions of the film. This reduction is attributed to the presence of MNPs disrupting the natural self-assembly of CNCs. The central and outer regions of the film retain a relatively strong peak with increasing UV-absorption for increasing MNP loading (Figure 9d).

Next, we evaluated the chiroptical properties of the magnetically patterned CNC/MNP films by using circular dichroism (CD) spectroscopy (Figure 10). First, a strong CD peak in CNC films dried without a magnetic field indicates the natural self-assembly of CNCs with a left-handed helical structure (Figure 10a).^21^ Furthermore, CD measurements were taken from both the center and the outer areas of the CNC/MNP films (Figure 10). Overall, increasing MNP concentration resulted in a significant red shift of the CD peak to 620 nm and a decrease in peak intensity at higher MNP loading and all rotational rates. These are attributed to the expansion of the pitch length and the reduced light transmission, both characteristic phenomena in CNC composites incorporating nanoparticles.^19^ The MNPs themselves do not exhibit any CD signals and thus do not contribute to the overall CD signal.^37,54^

**Figure 10:**
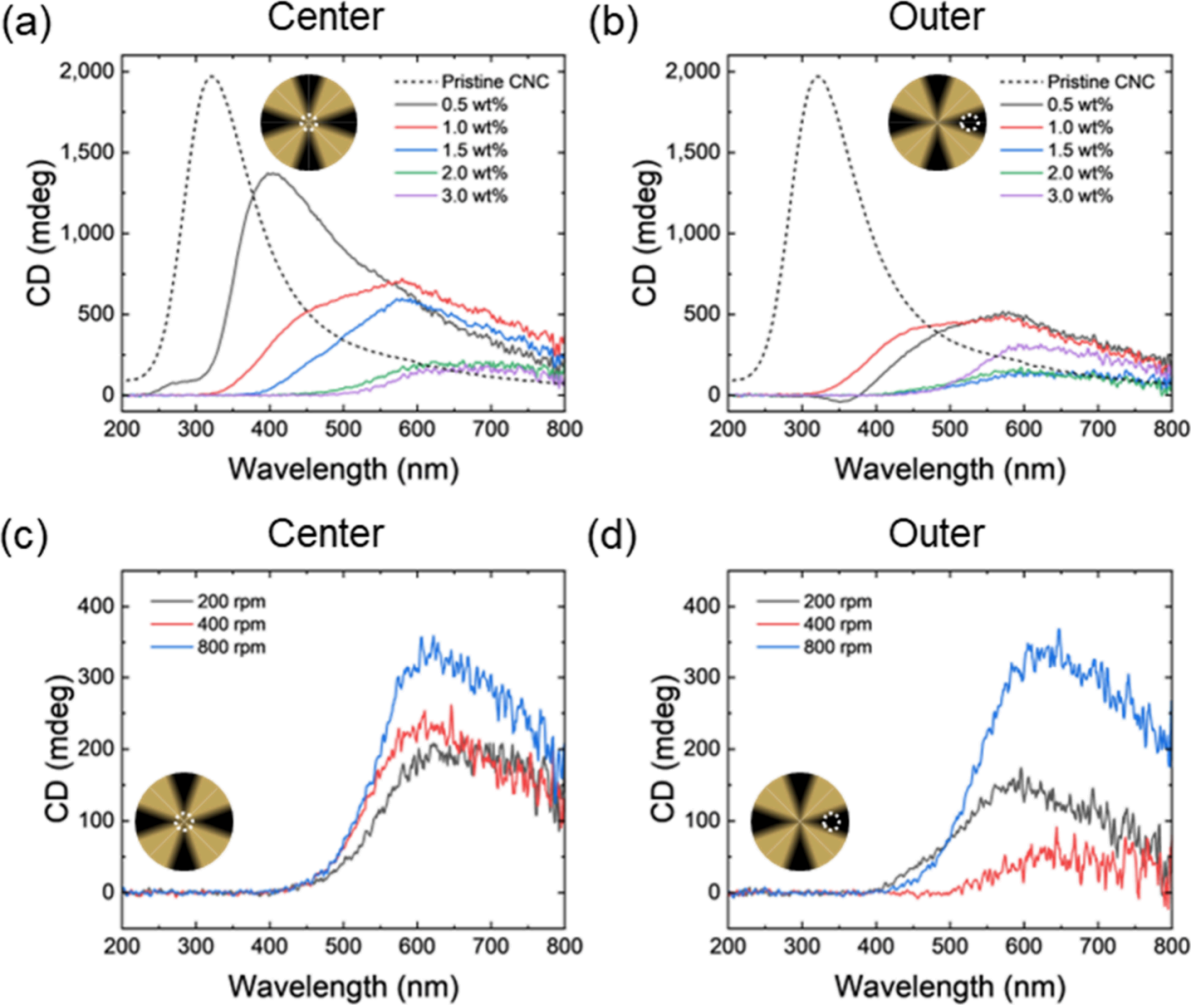
Circular dichroism (CD) spectra of patterned CNC/MNP composite films: (a, c) the center of the films and (b, d) the outer area of the films. Insets show the positions of local spots for CD measurements. MNP loading for the samples in (c) and (d) was 2.0 wt %.

Next, CNC/MNP composite films were prepared using rotational magnetic fields with varying rotational directions, clockwise and counterclockwise, to investigate whether changes in shearing direction influence the symmetry of the optical texture (Figure 11 and Figure S29). Remarkably, drastically different pinwheel optical patterns were observed around the center of the composite films depending on the rotational direction in C-POM micrographs (Figure 11b,f,c,g). To further elucidate changes in the helical organization, CD spectra signals were collected for both specimens in different locations (Figure 11d,h).

**Figure 11:**
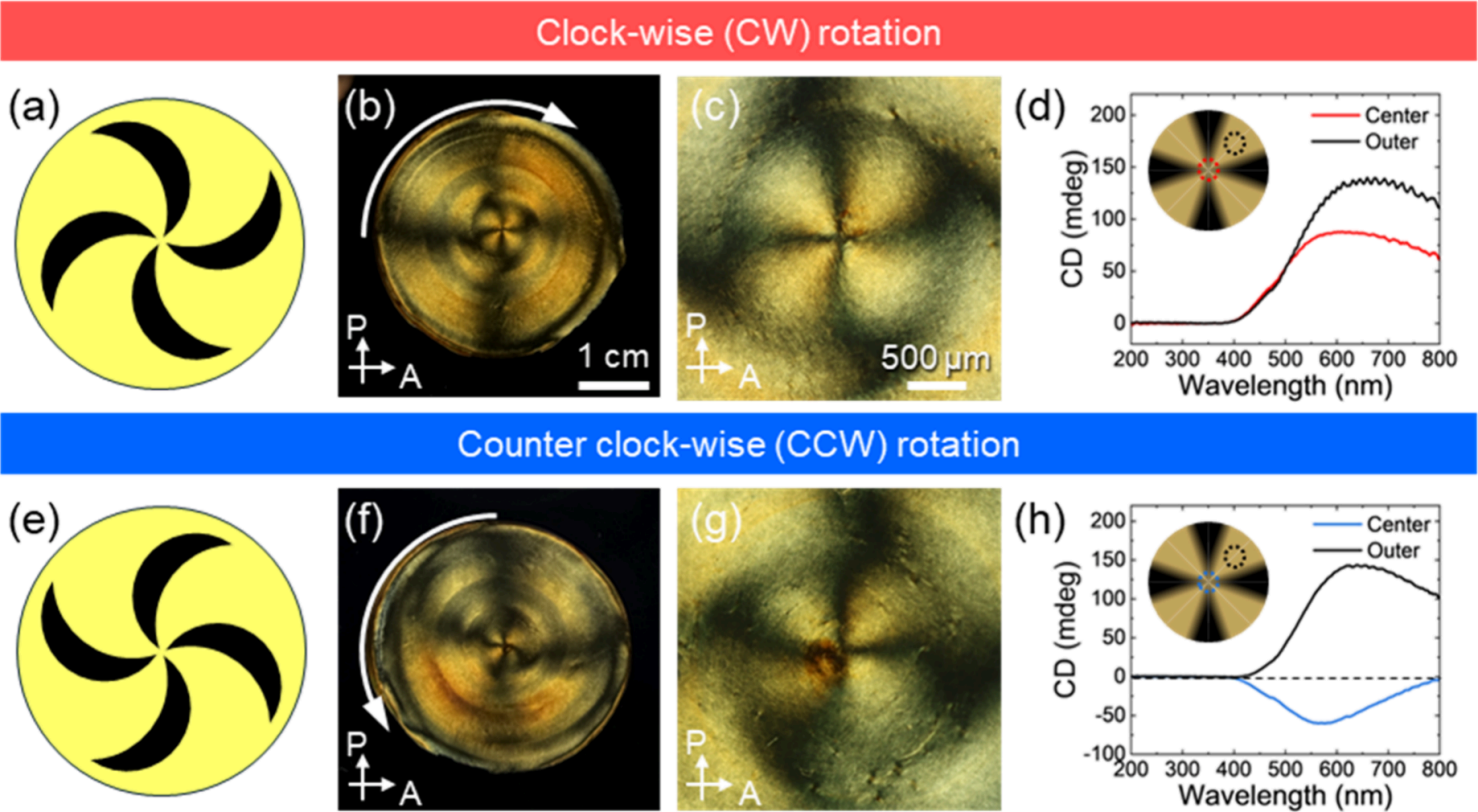
CNC/MNP composite films formed under rotational magnetic fields with different rotational directions. (a, e) Schematic illustration of pinwheel patterns around the center of CNC/MNP composite films. (b, f) Entire and (c, g) enlarged C-POM micrographs of CNC/MNP composite films with different magnetic rotational directions. (d, h) CD signals at the center of CNC/MNP composite films. Scale bar in (b) shares with (f) and in (c) shares with (g).

A rotating magnetic field generates vortices at the center of the solution, which influence the helical organization of CNCs. By altering the direction of the rotating magnetic field, it is possible to control the handedness of the helical organization of CNCs. A clockwise rotation of the magnetic field resulted in the left-handed chiroptical pattern, as confirmed by the positive CD signals (Figure 11d). However, when the direction of the magnetic field was switched to counterclockwise, the CD signal reverts to negative, reflecting a right-handed pattern (Figure 11h). Multiple measurements with different angles confirmed that negative CD signals are not artifacts from the instruments (Figure S30). In addition to CD measurements, SEM micrographs also show their reflecting handedness in both macro- and microscales (Figure S31). Their crack directions are opposite, the upper left direction and upper right direction, respectively, which are typical left-handed and right-handed liquid crystalline structures. CNC bundles also rotate left-handed in the composite film evaporated under a clockwise rotational magnetic field, while an opposite rotational magnetic field induces right-handed rotation of CNC bundles. CD flipping of CNC positive to negative value induced by handedness inversion, left- to right-handed, which is confirmed by previous literature studies, including shear-printing,^18^ magnetic field gradient,^37^ and rotation of solution during evaporation.^55^ This reversal in the CD sign indicates that the chirality of the rotational pattern switched to right-handed helicity, as driven by the opposite rotational flow direction applied during evaporation-driven assembly.

## Conclusions

In conclusion, we have presented a scalable method for the precise assembly of needle-like cellulose nanocrystals into large-scale azimuthal patterns on the centimeter scale using rotational magnetic fields during suspension evaporation. The rotational magnetic field generates a dynamic shear force that controls the assembly behavior, inducing unique local monodomain orientational patterns with high local order approaching the theoretical limit (0.96 ± 0.02). This shear-induced flow results in azimuthal organization and distinct chiral optical signatures, including large (centimeter)-area Maltese cross patterns under crossed polarizers. Notably, programmable helicity can be reversed from left-handed to right-handed organization by changing the direction of the rotational magnetic field during induced assembly.

The improvement of the consistency and quality of the azimuthal orientation could further result in the expansion of the spatial scale of the signature of optical textures, as preprogrammed by the rotational magnetic field. We suggest that this approach can be extended to other anisotropic nanostructures, even those without inherent chirality, enabling the creation of large-scale monodomain thin films with tailored chiroptical properties and magnetically controlled optical texture. The approach suggested here can be utilized for scalable fabrication for large-area bioderived chiral photonic materials with preprogrammed optical textures.

## Experimental Section

### Synthesis of Cellulose Nanocrystals

CNCs were prepared by sulfuric acid hydrolysis of wood pulp according to the established procedure.^21^ After rinsing and drying the pulp, 34 g of dried wood pulp was dispersed into 600 mL of 64 wt % sulfuric acid (aqueous) at 45 °C and mixed using a magnetic stirrer for 90 min. The suspension was added to deionized water, which is 5-fold the amount of the suspension. Then, the diluted suspension was put in an ambient state overnight for the sedimentation of cellulose, and the supernatant was removed. The remaining cellulose was washed with centrifugation three times. The suspension was purified using dialysis membranes in deionized water for 3 days with a daily exchange of water, until the pH value of the water outside became constant. The suspension was centrifuged twice, the supernatant was kept, and the pellet was sonicated. The CNC suspension was stored at room temperature.

### Synthesis of Magnetic Nanoparticles, Fe_3_O_4_

MNPs were synthesized according to an established method.^35^ Under a N_2_ atmosphere and at 70 °C, a round-bottom flask was charged with FeCl_2_·4H_2_O (5 mmol) and FeCl_3_·6H_2_O (10 mmol) into 50 mL of deionized water and stirred for 30 min. Six mL of ammonium hydroxide NH_3_(aq) was added, and the system was stirred for another 30 min. A 1 mL portion of 1.5 g/mL citric acid solution was added to the system, and the mixture was stirred for 120 min under a N_2_ atmosphere at 70 °C. The resulting solution was cooled to room temperature and then washed with deionized water and filtered by centrifugation three times (10,000 rpm, 10 min round). From TEM images, the average size of MNPs was evaluated as 7.0 ± 2.3 nm.

### Preparation of CNC/MNP Composite Film

For mixed suspension, 0.2 wt % of MNP suspensions was added to 5 wt % of 2 mL CNC suspension under stirring using a micropipet drop by drop with the desired amount for control of Fe_3_O_4_ concentration, 0.5, 1.0, 1.5, 2.0, 3.0, and 5.0 wt %, respectively. The composite suspensions were vigorously stirred overnight without a lid. The suspensions with a concentration of over 1.0 wt % were dried until the desired amount of suspension was obtained by vigorous stirring. The total volume of the composite suspension was around 2.5 mL. Then, suspensions were cast in the Petri dish with a 3.5 cm diameter and dried at different stirring rates (0, 200, 400, and 800 rpm). The thickness of films is in the range of 46 to 47 μm.

### Optical Microscopy (OM)

Optical microscope images were obtained with an Olympus BX51 microscope. The images were taken in transmission mode under bright field conditions to characterize the appearance and uniformity of the samples. To observe the optical appearance, the crossed polarizer was attached to the bottom of the stage.

### Scanning Electron Microscopy (SEM)

Surface and cross-sectional SEM images of CNC/MNP composite films were obtained with a Hitachi SU8230 microscope. All films were sputtered with gold before measurements.

### Circular Dichroism (CD) Measurements

CD was performed using a Jasco J-815 CD spectropolarimeter. The film is cut to 2 mm × 2 mm × 46 μm dimensions and then mounted to the sample holder with two quartz slides to measure the CD at local points.

### Tensile Test

The tensile test was conducted with a universal tensile machine (EZ-SX, Shimadzu). Each sample was cut into rectangular specimens of 2 × 10 × 15 mm. Elongation rate for tensile measurements was 50 mm/min.

### Atomic Force Microscopy (AFM)

Surface morphologies are observed by AFM (Dimension Icon, Bruker) in the standard tapping mode.^56^ Scans of the composite films are conducted by using an 8 nm radius regular tip. The size of the images is 1024 × 1024 pixels. The orientational image analysis was conducted with GTFiber software.^18,41^

### Magnetic Field Simulation

The vectors of the magnetic field and magnetic flux density were simulated by Ansys Maxwell software. The geometry and magnetic properties of the simulated magnets are based on the permanent magnets, ferrite magnets, arranged on a hot plate stirrer. The simulated magnetic field direction was taken at a plane 2 cm above the magnet, with air set as the surrounding medium. The geometry of the magnet was drawn in Figure 1d. The magnetic poles are directed in a longer direction.

## Supplementary Material





## Notes

Optical and crossed polarized optical images/micrographs of CNC/MNP composite films, AFM topographies of CNC/MNP composite film surface, orientational order parameter from AFM images, cross-section SEM micrographs of CNC/MNP composite films with different MNP loading, FT-IR spectra of MNPs, mechanical properties of CNC/MNP composite films, images of the custom magnetic setup, and CD spectra (PDF)
CNC/MNP solution with 2.0 wt % MNP loading on the magnetic stirrer (MP4)
