# Supplementary material for: Spatially Programmable Chirality in Cellulose Nanocrystal Films via Rotational Magnetic Flow
Source: ACS Appl Mater Interfaces. 2025 Aug 30;17(36):51022–31. doi: 10.1021/acsami.5c12750 (PMC12442017; doi:10.1021/acsami.5c12750)
Supplement: Supplementary file 1 [file am5c12750_si_001.pdf]

Supporting Information

Spatially Programmable Chirality in Cellulose Nanocrystal Films via Rotational Magnetic Flow

Jisoo Jeon[1], Dhriti Nepal[2], Michael E. McConney[2], Timothy J. Bunning[2], Vladimir V. Tsukruk[1]*

[1] School of Materials Science and Engineering, Georgia Institute of Technology, Atlanta, GA 30332, USA

[2] Air Force Research Laboratory, Wright-Patterson Air Force Base, Ohio 45433, United States

*Corresponding author e-mail: vladimir@mse.gatech.edu

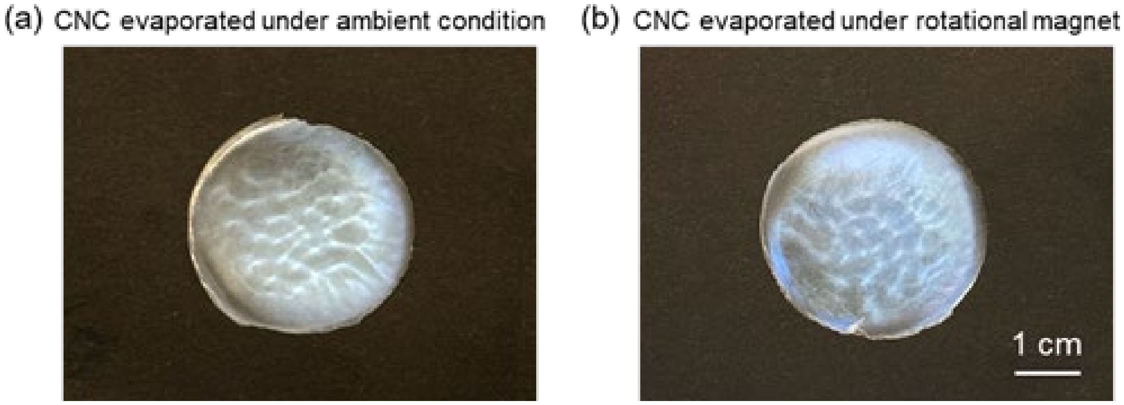

Figure S1. Photos of neat CNC film evaporated under (a) the ambient condition and (b) rotational magnetic field. Applied rotational rate was 100 rpm.

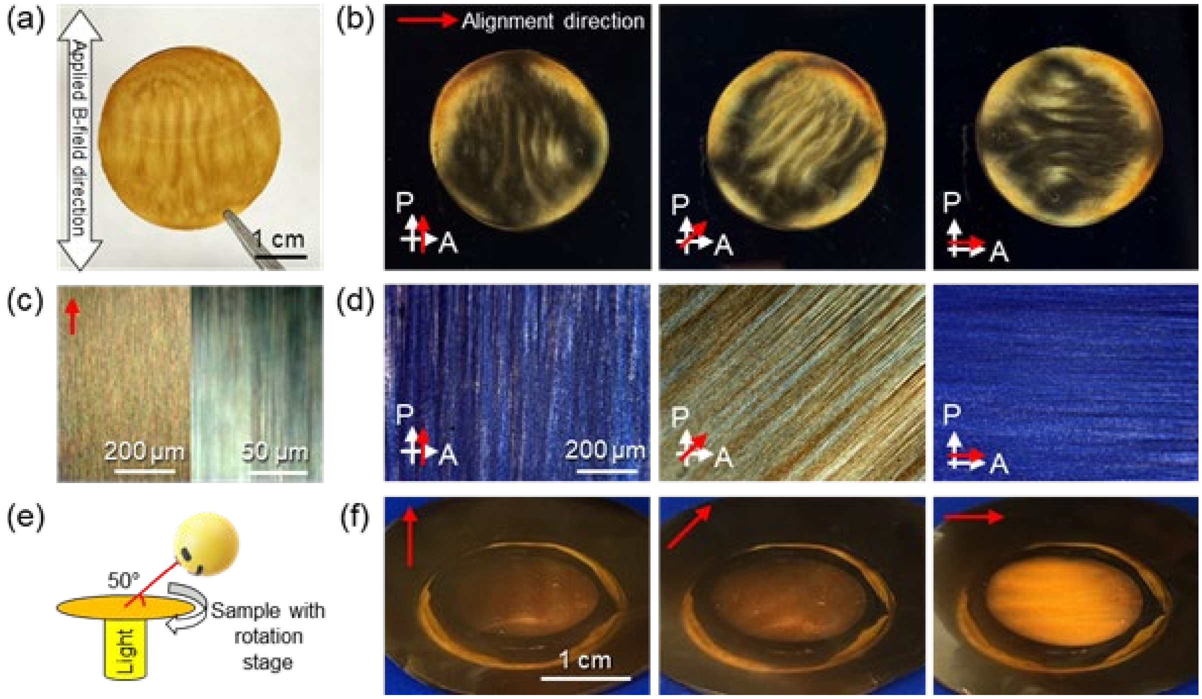

Figure S2. (a) Photo, (b) crossed polarized optical images, (c) optical, and (d) crossed polarized optical micrographs of CNC/MNP composite film evaporated under a static unidirectional magnetic field. (e) Schematic illustration of the light scattering observation with an incident angle. (f) Angular-dependent light scattering observed at an incident angle of 50°. Scale bar in (a) shares with images in (b).

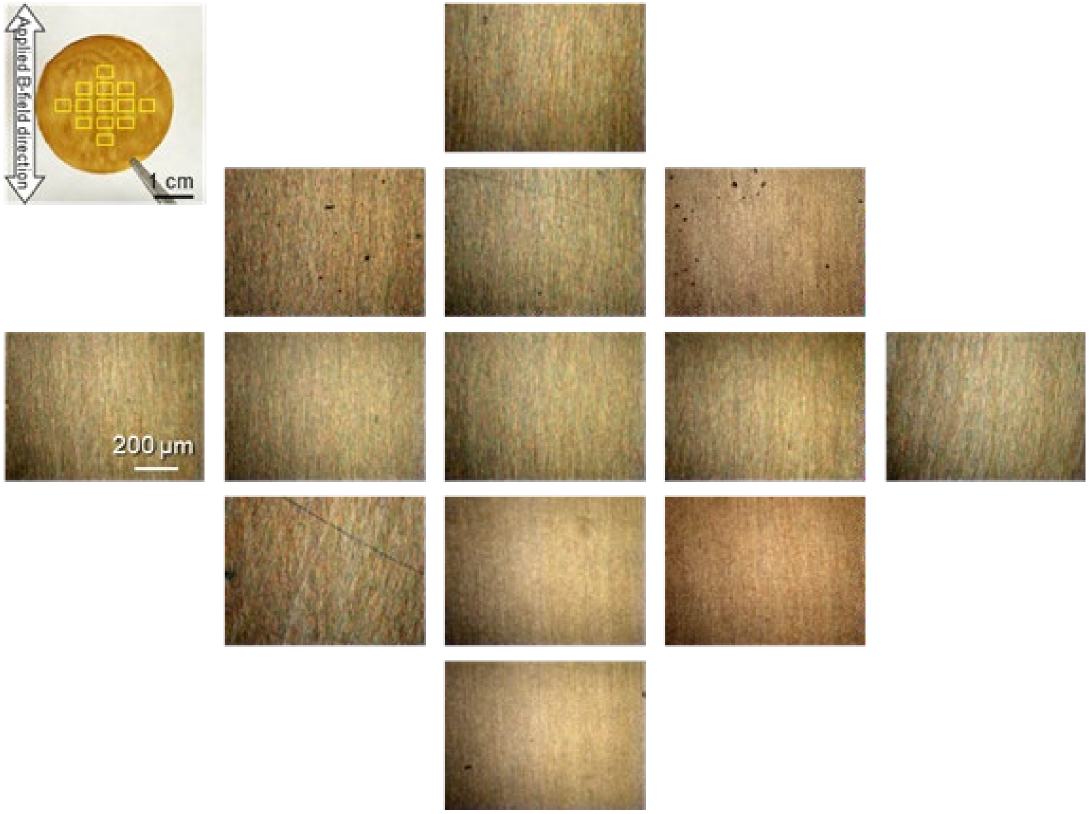

Figure S3. Optical micrographs at each position in the inset photo. The position of yellow boxes in the insect photo coincides with the position of optical micrographs.

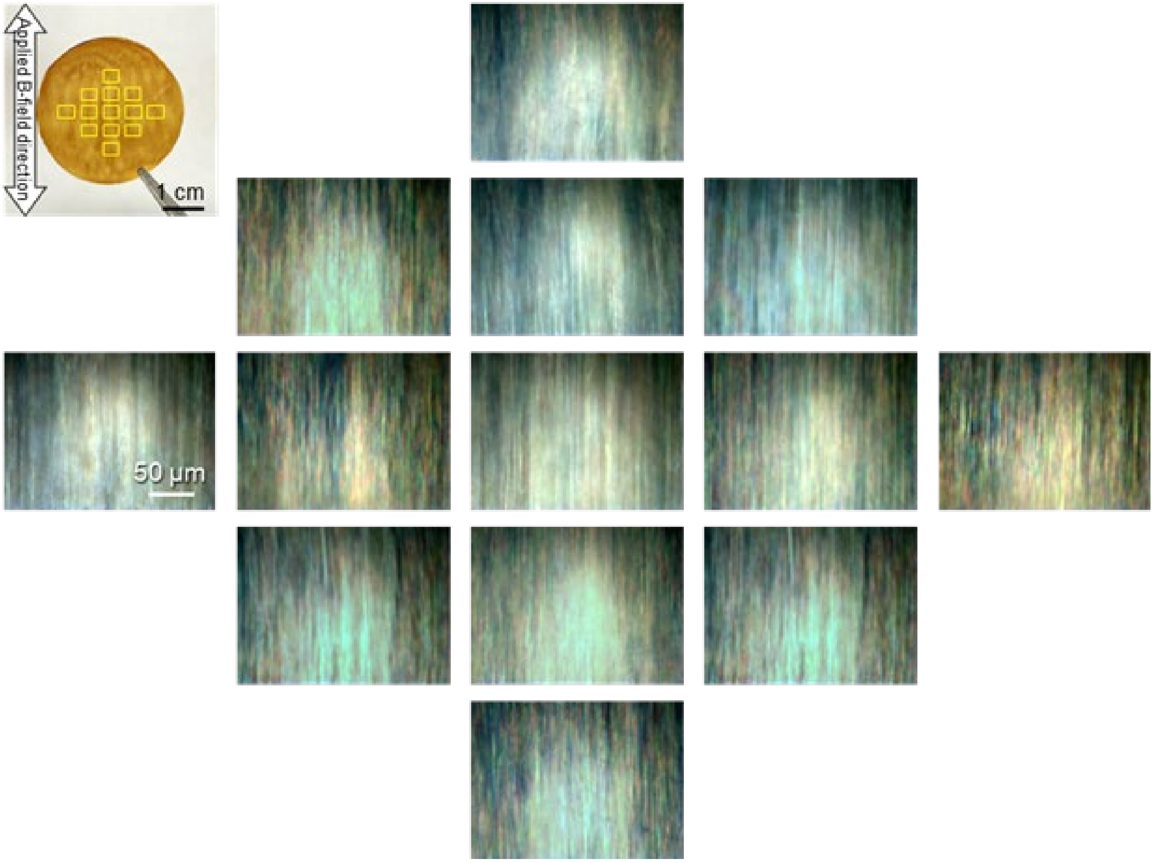

Figure S4. Optical micrographs at each position in the inset photo. The position of yellow boxes in the insect photo coincides with the position of optical micrographs.

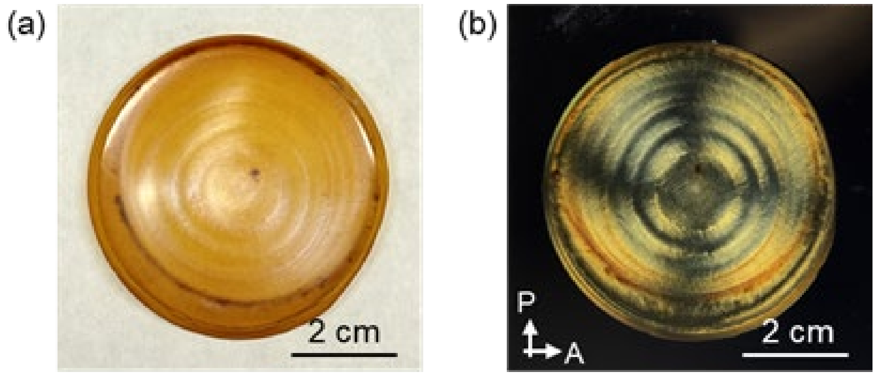

Figure S5. (a) Optical and **(b)** crossed polarized image CNC/MNP composite film evaporated in a 55 mm diameter Petri dish. Rotation rate: 100 rpm. MNP loading in film: 2.0 wt%.

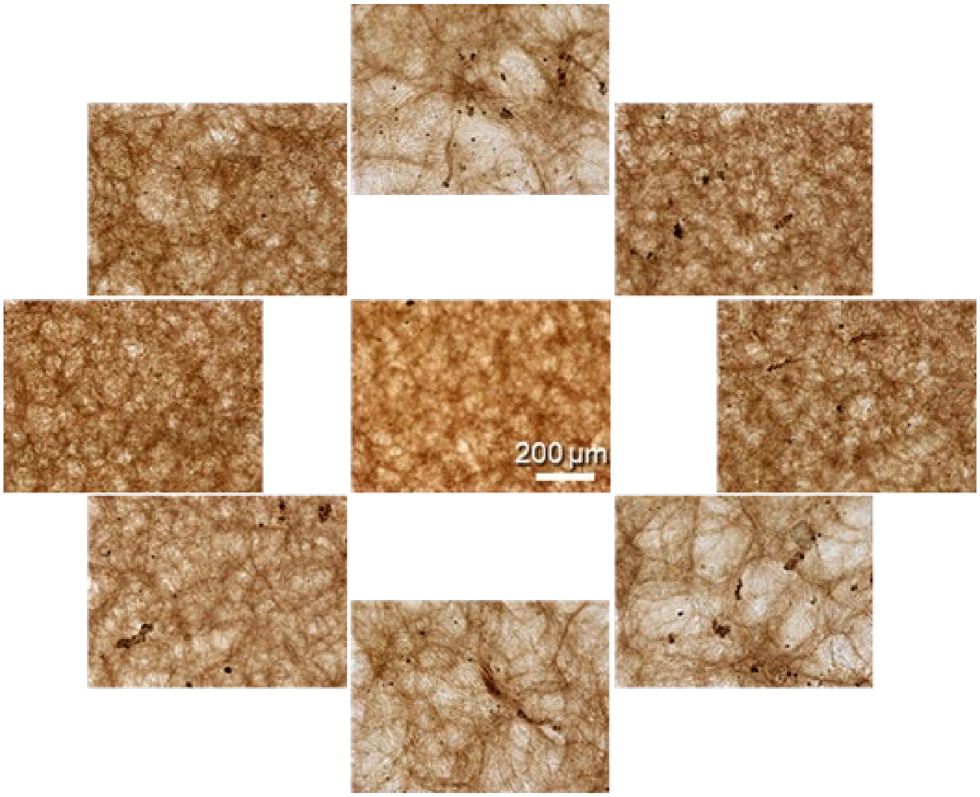

Figure S6. Optical micrographs of surface morphologies of CNC/MNP composites films obtained without rotational magnetic field. Scale bar is the same for all images.

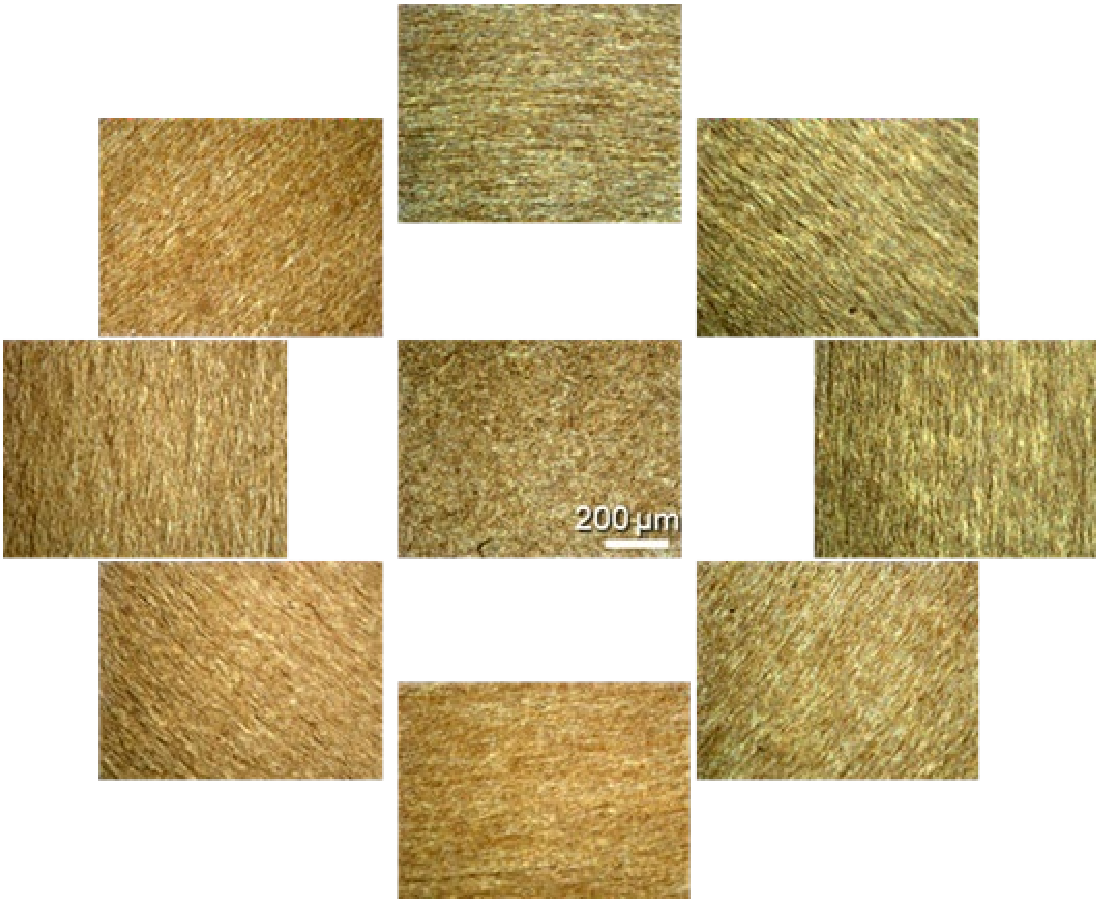

Figure S7. Optical micrographs of surface morphologies of CNC/MNP composites films obtained under rotational magnetic field. Scale bar is the same for all images.

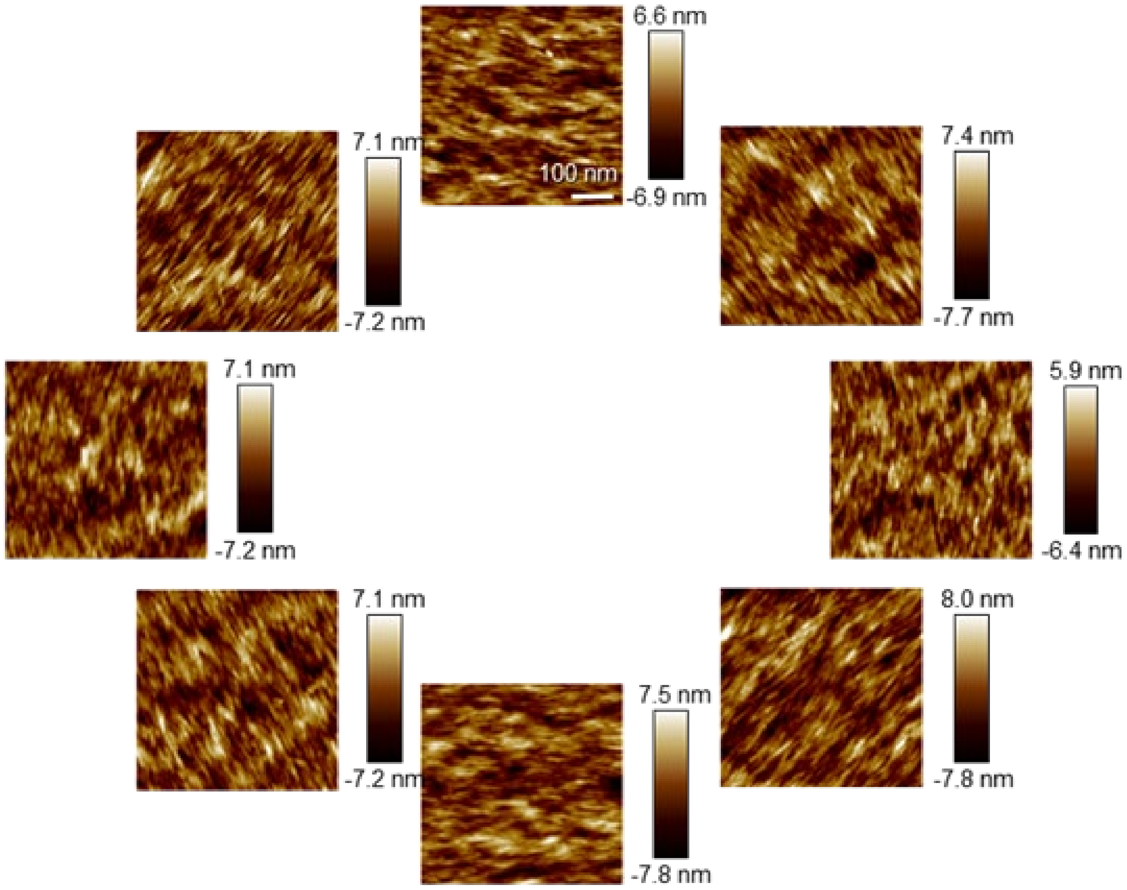

Figure S8. AFM topographies of surface of CNC/MNP composites films obtained under rotational magnetic field. Scale bar is the same for all images.

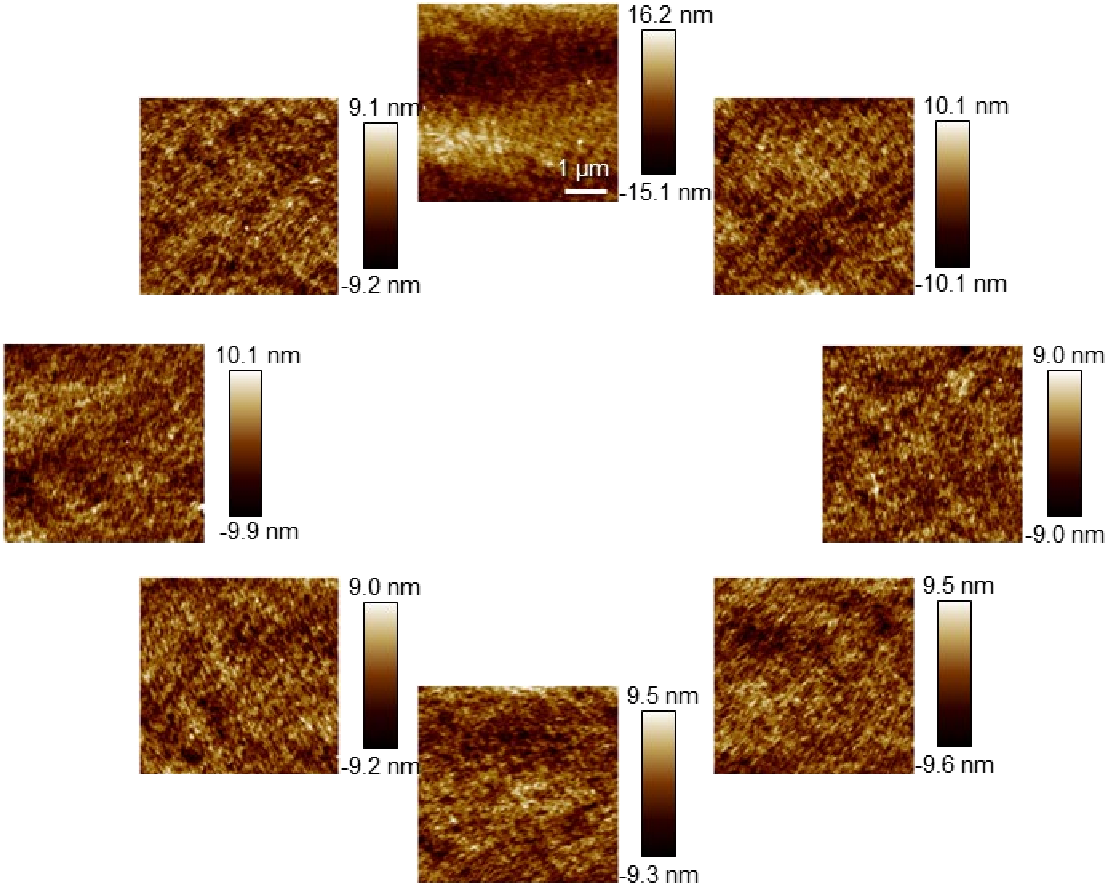

Figure S9. AFM topographies of surface of CNC/MNP composites films obtained under rotational magnetic field. Scale bar is the same for all images.

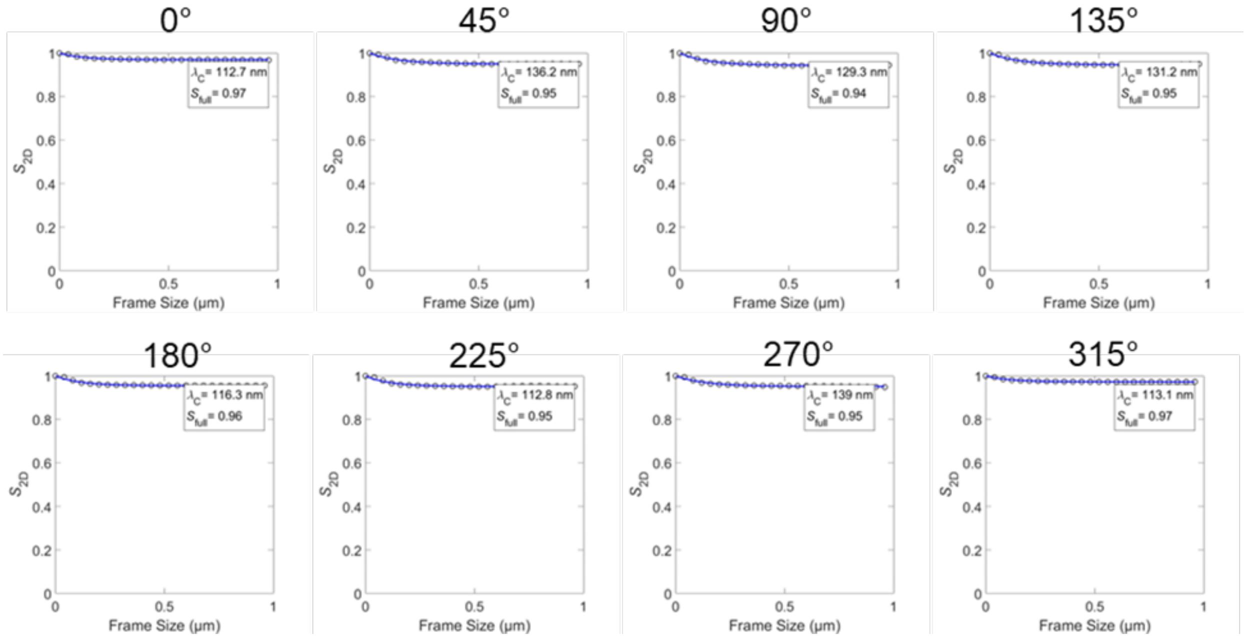

Figure S10. Orientational order parameter from AFM image analysis for different directions and at different scales.

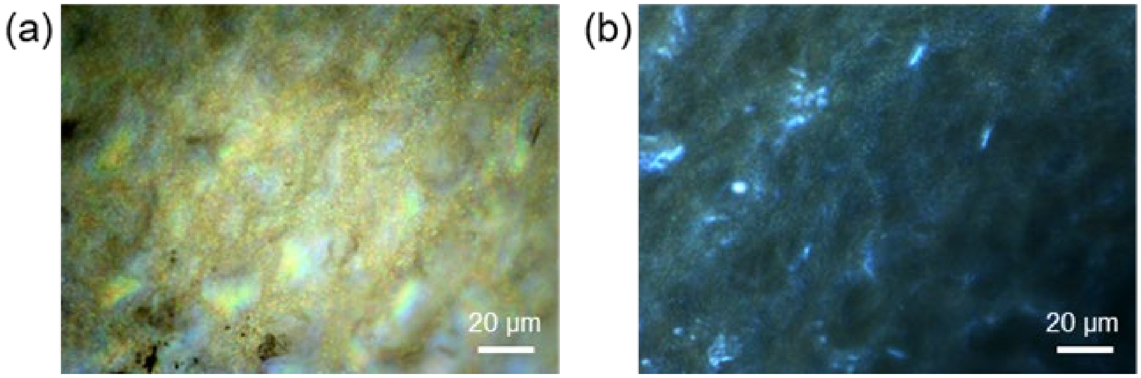

Figure S11. (a) Bright field and **(b)** dark field optical microscopy of CNC/MNP composite film with 2.0 wt% of MNPs.

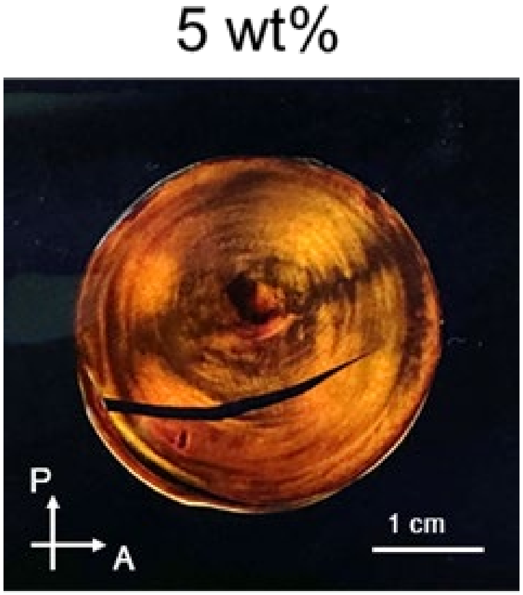

Figure S12. C-POM image of CNC/MNP composite films consisting 5 wt% MNPs obtained under rotational magnetic field. Rotational rate was 200 rpm.

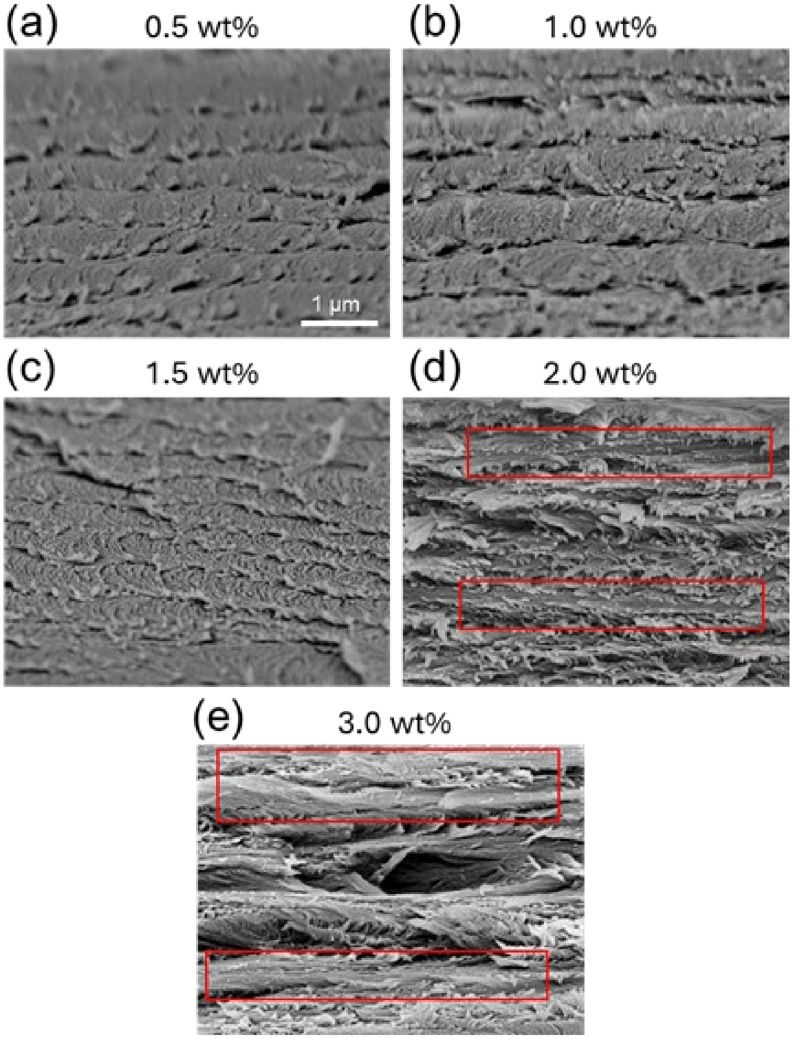

Figure S13. SEM micrographs of cross-section of CNC/MNP composite films with different MNP concentration. (a) 0.5 wt%, (b) 1.0 wt%, (c) 1.5 wt%, (d) 2.0 wt%, (e) 3,0 wt%. All the SEM images share the scale bar in (a).

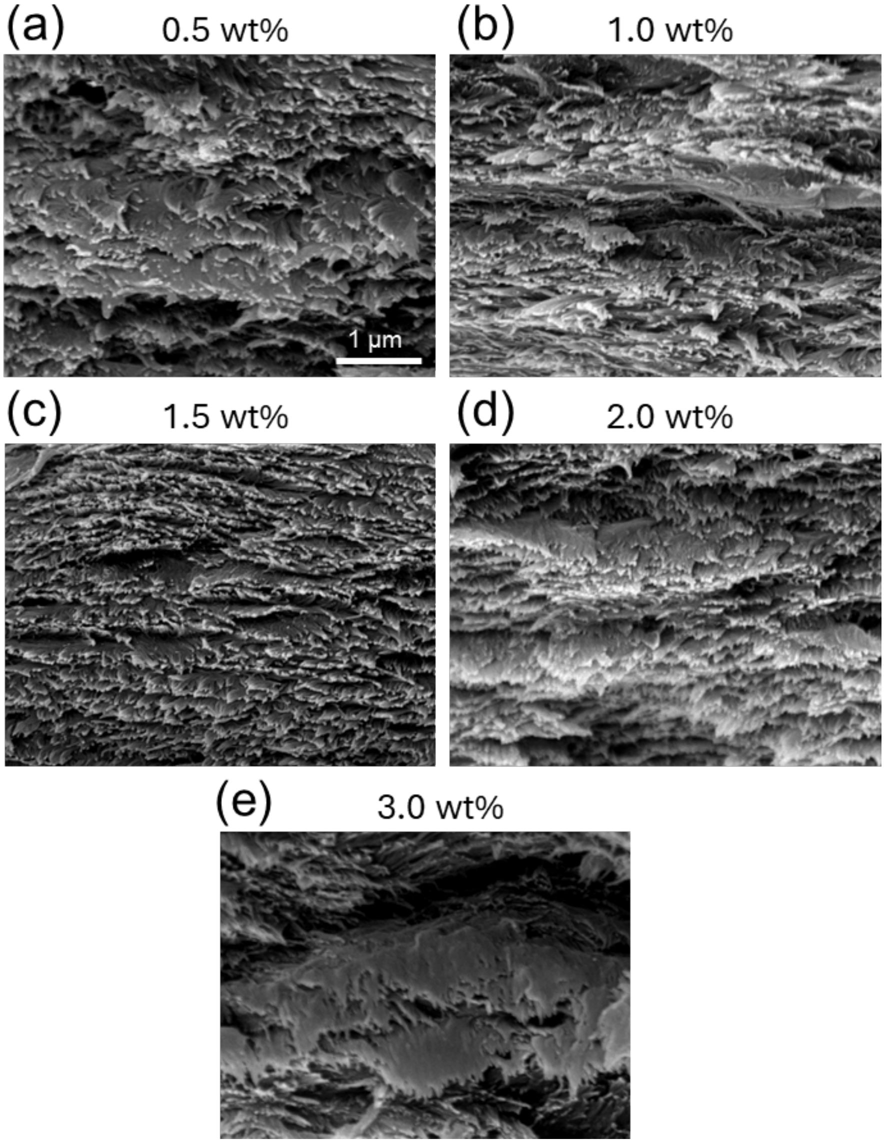

Figure S14. SEM micrographs of cross-section of CNC/MNP composite films with different MNP concentration the axial direction of the film. (a) 0.5 wt%, (b) 1.0 wt%, (c) 1.5 wt%, (d) 2.0 wt%, (e) 3,0 wt%. All the SEM images share the scale bar in (a).

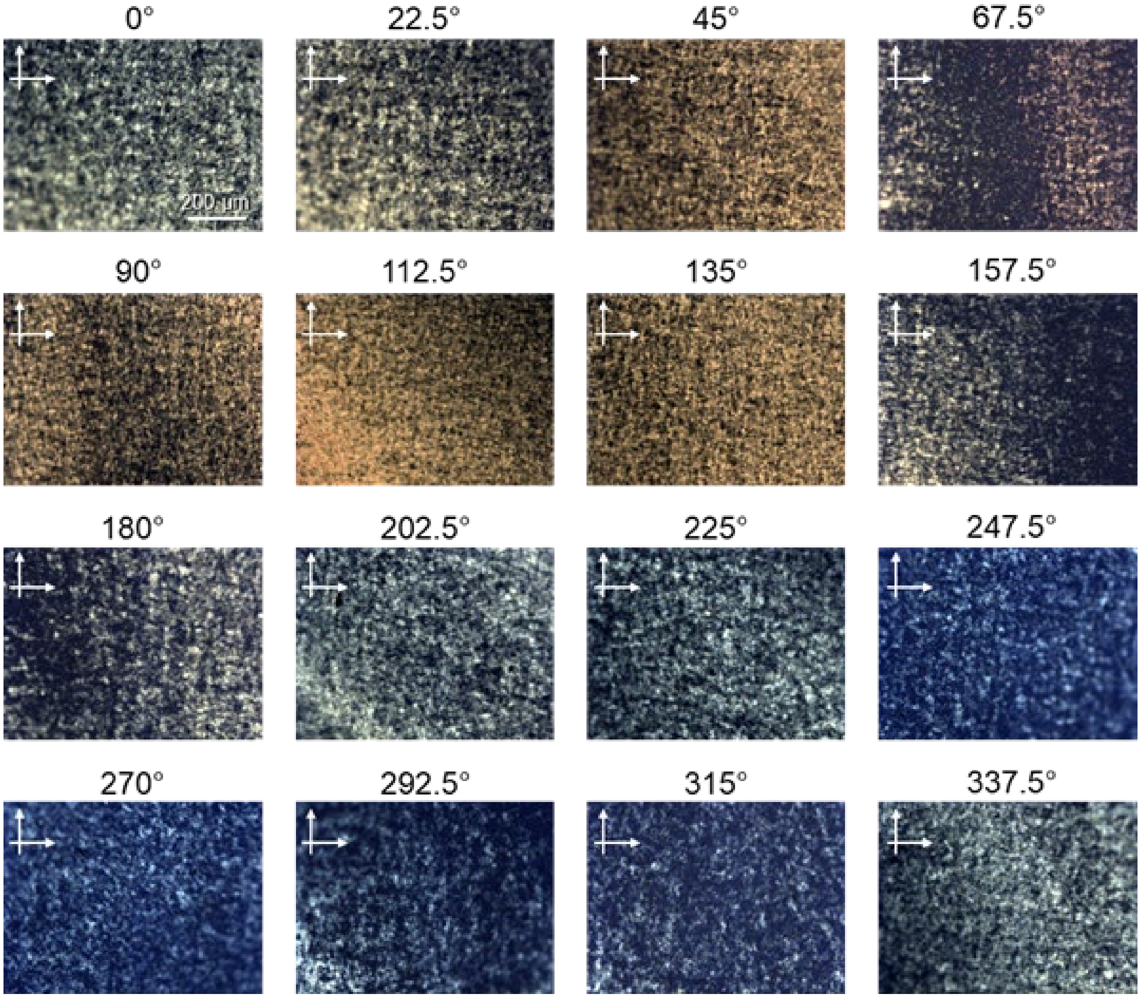

Figure S15. C-POM micrographs according to the position with angles of CNC/MNP composites film with 2 wt% of MNPs obtained under 200 rpm rotational magnetic field. All the images share the scale bar.

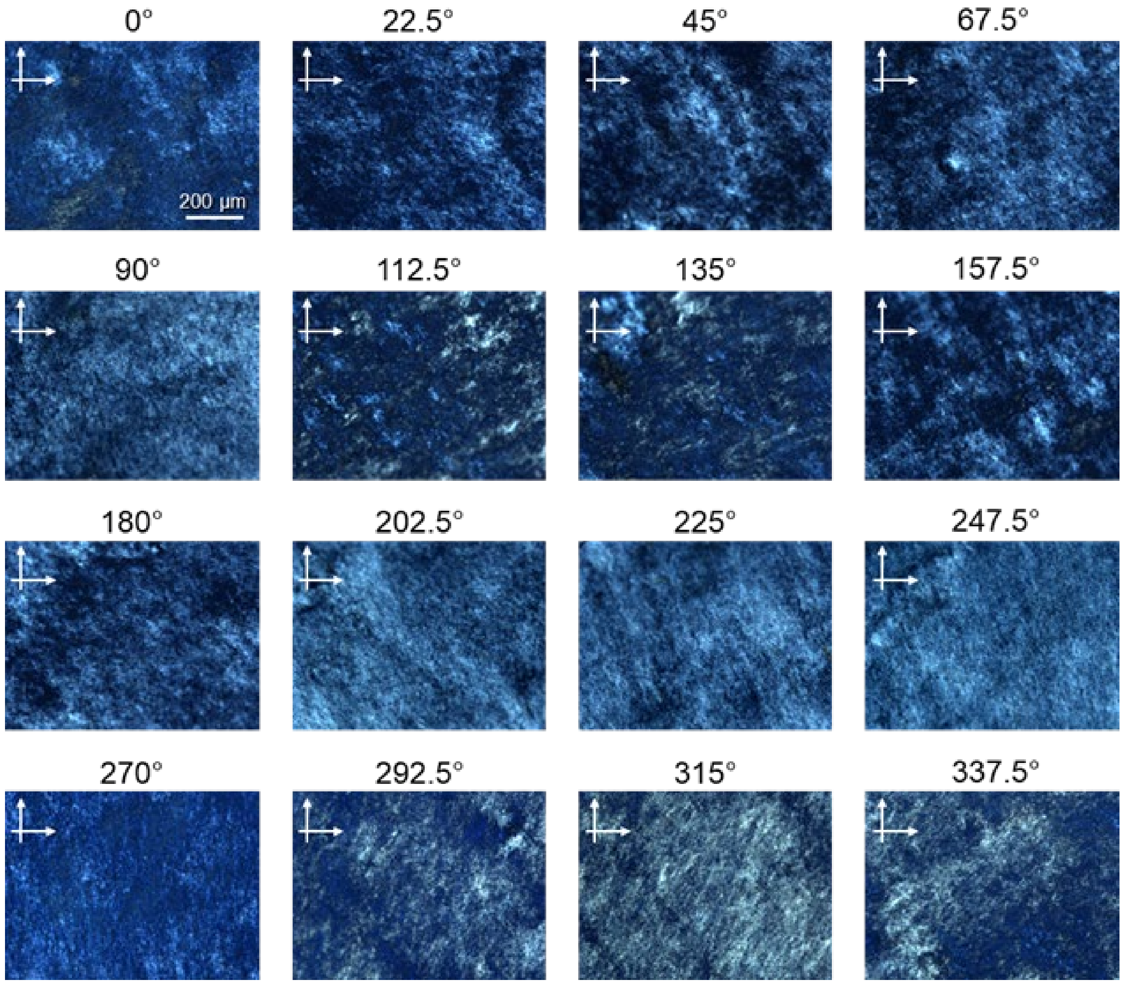

Figure S16. C-POM micrographs according to the position with angles of CNC/MNP composites film with 0.5 wt% of MNPs obtained under 200 rpm rotational magnetic field. All the images share the scale bar.

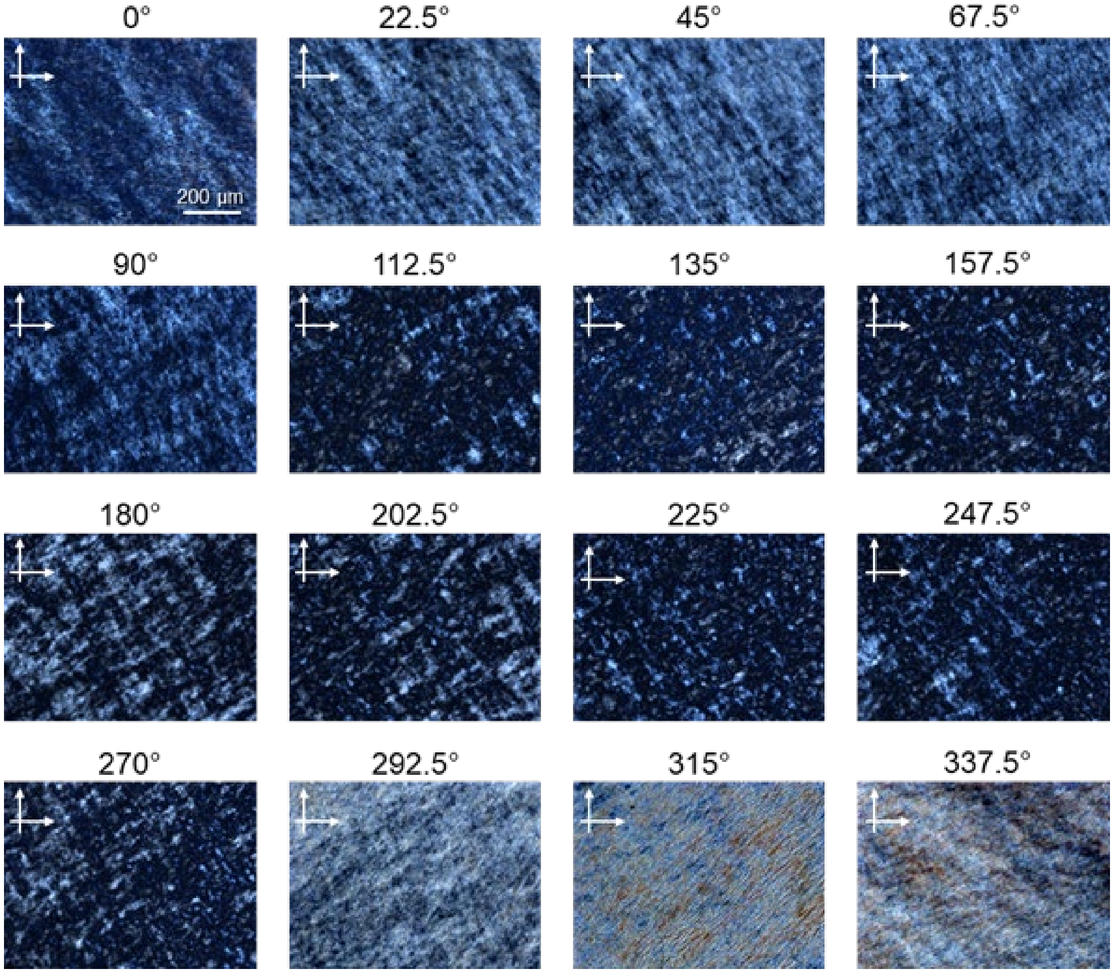

Figure S17. C-POM micrographs according to the position with angles of CNC/MNP composites film with 1.0 wt% of MNPs obtained under 200 rpm rotational magnetic field. All the images share the scale bar.

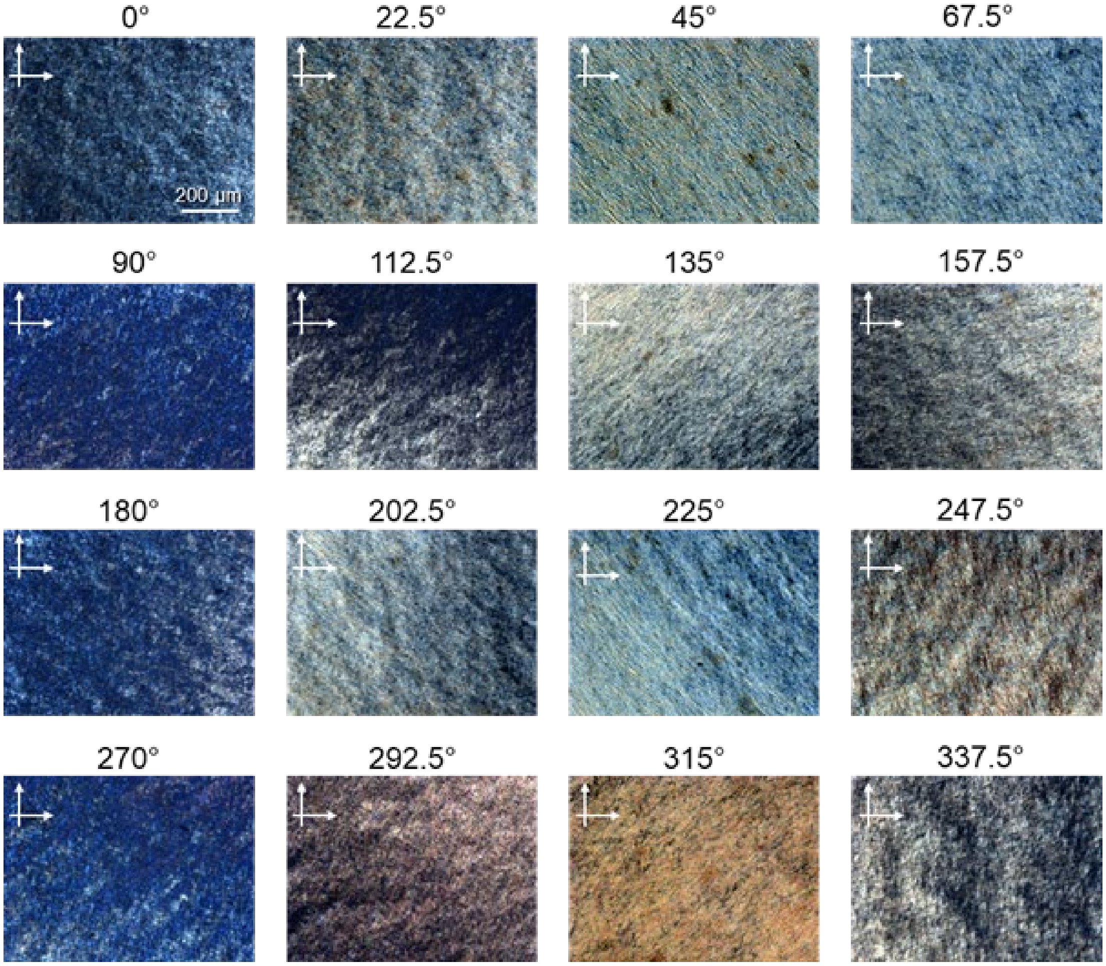

Figure S18. C-POM micrographs according to the position with angles of CNC/MNP composites film with 1.5 wt% of MNPs obtained under 200 rpm rotational magnetic field. All the images share the scale bar.

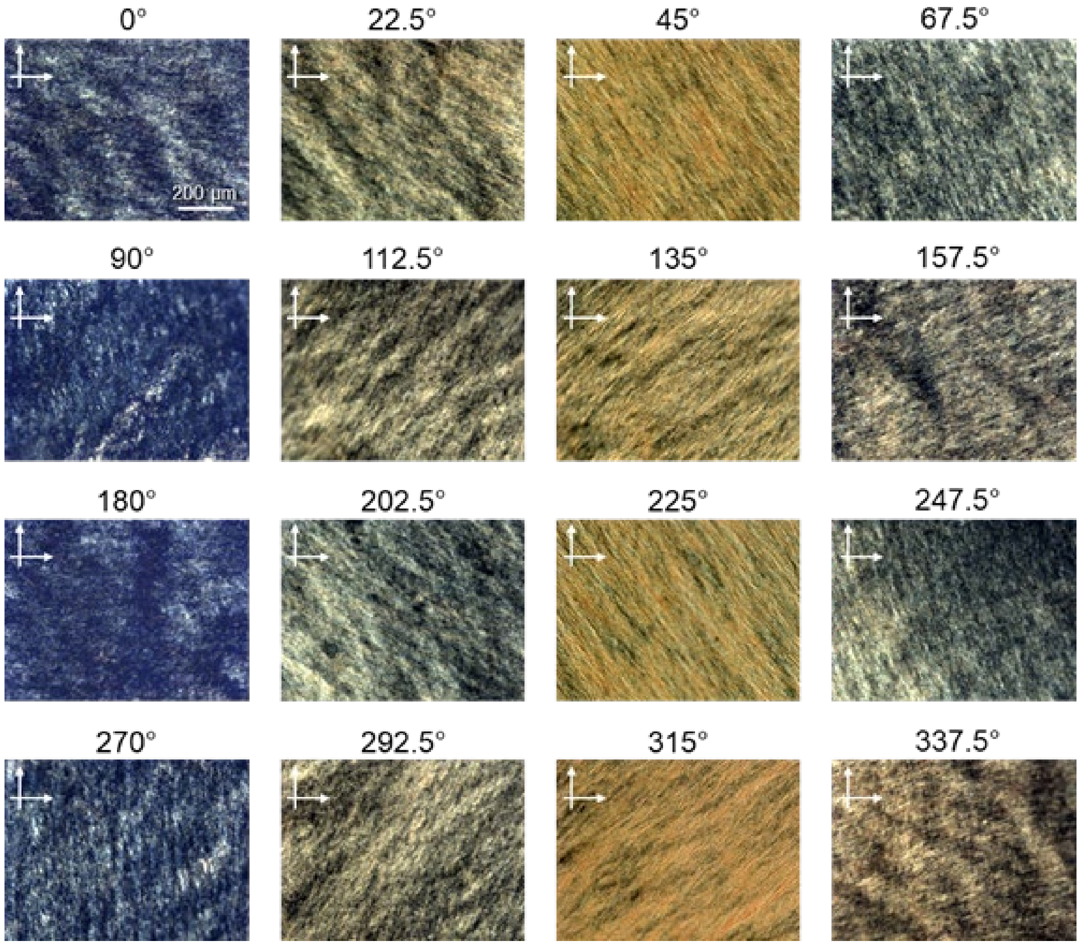

Figure S19. C-POM micrographs according to the position with angles of CNC/MNP composites film with 2.0 wt% of MNPs obtained under 200 rpm rotational magnetic field. All the images share the scale bar.

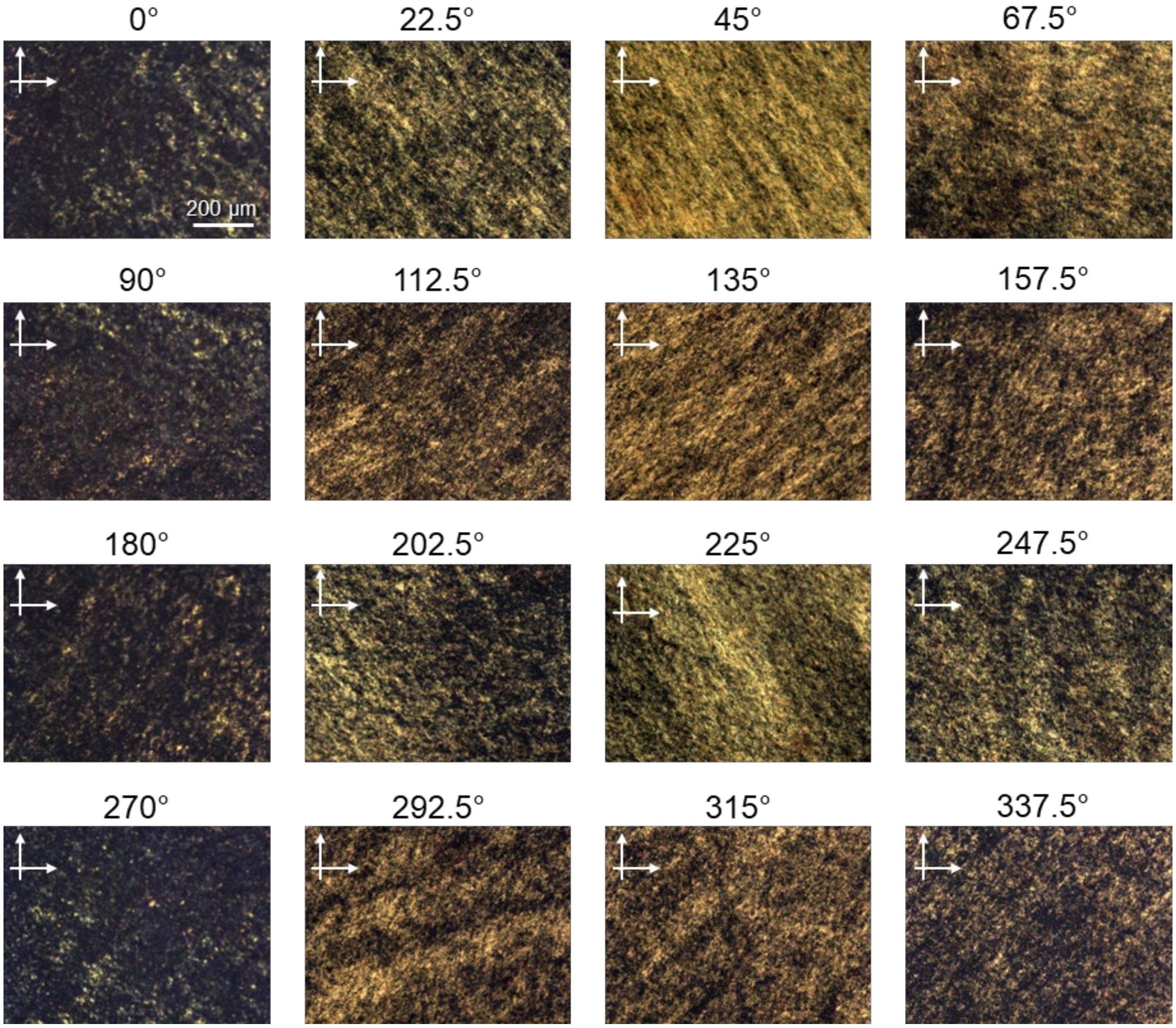

Figure S20. C-POM micrographs according to the position with angles of CNC/MNP composites film with 3.0 wt% of MNPs obtained under 200 rpm rotational magnetic field. All the images share the scale bar.

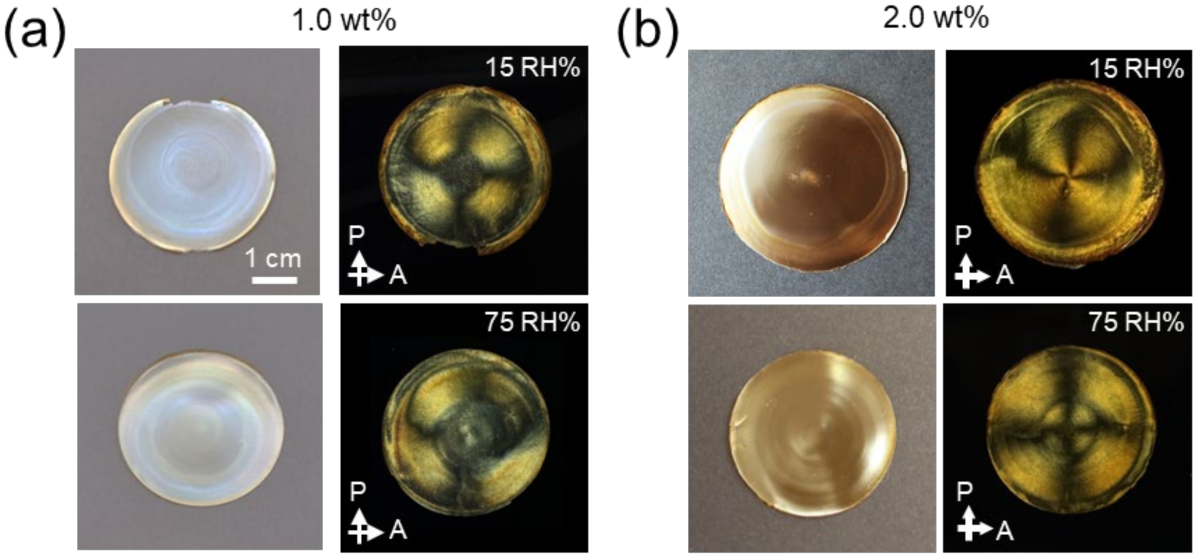

Figure S21. Images of reproducible concentric patterns of CNC/MNP composite films obtained under rotational magnetic field with different relative humidities. (a) 1.0 wt% and (b) 2.0 wt% of CNC concentrations. All the images share the scale bar.

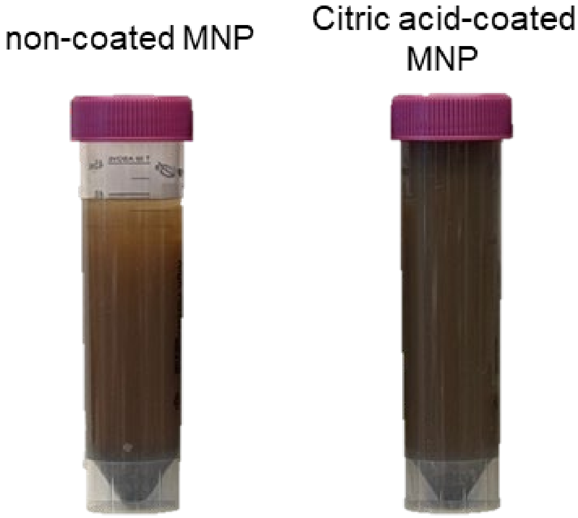

Figure S22. MNP aqueous solutions with/without surface modification. (left) non-coated MNPs, (right) citric acid-coated MNPs.

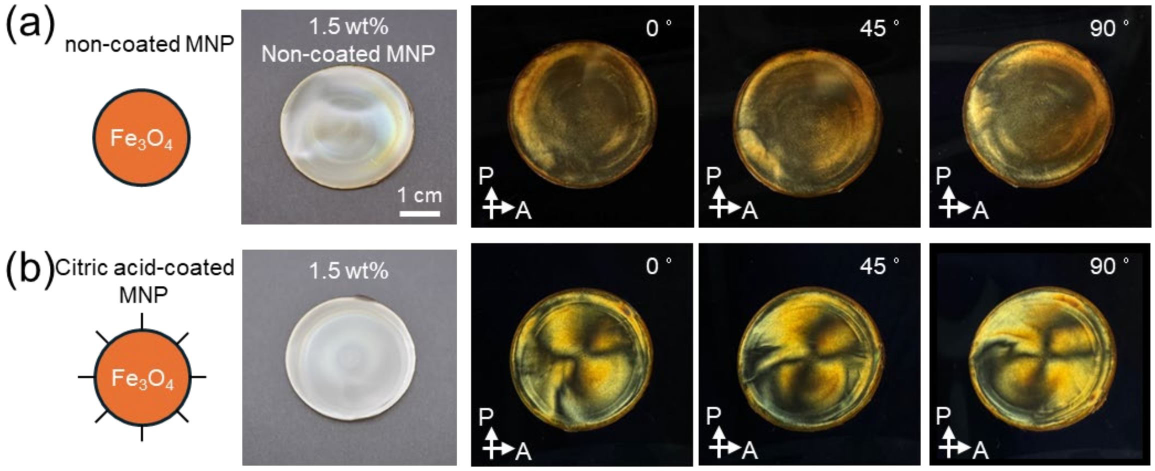

Figure S23. Schematic illustrations of MNPs (a) without or (b) with surface modification. (Grey background) Images of CNC/MNP composite films obtained under rotational magnetic field. (Black background) C-POM images of CNC/MNP composite films dried under rotational magnetic field. All the images share the scale bar.

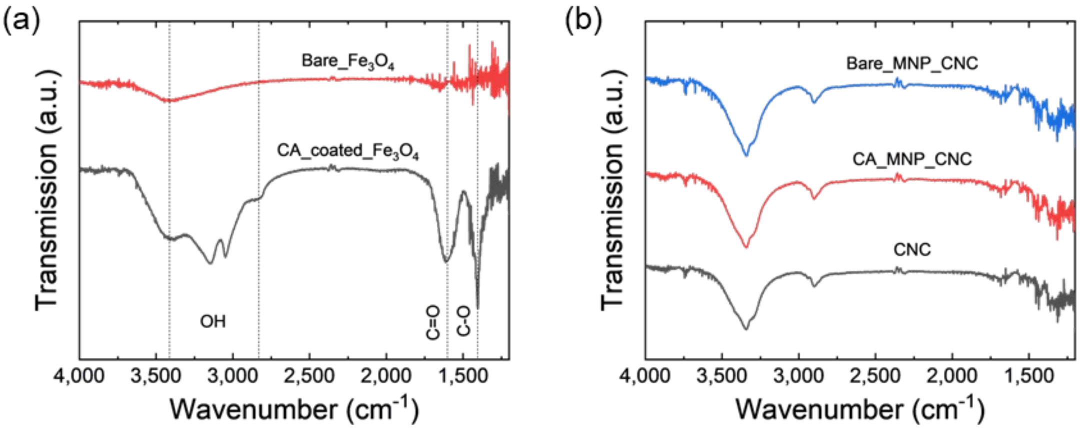

Figure S24. FT-IR spectra of (a) MNPs with/without citric acid (CA) coating, and (b) CNCs with/without MNPs.

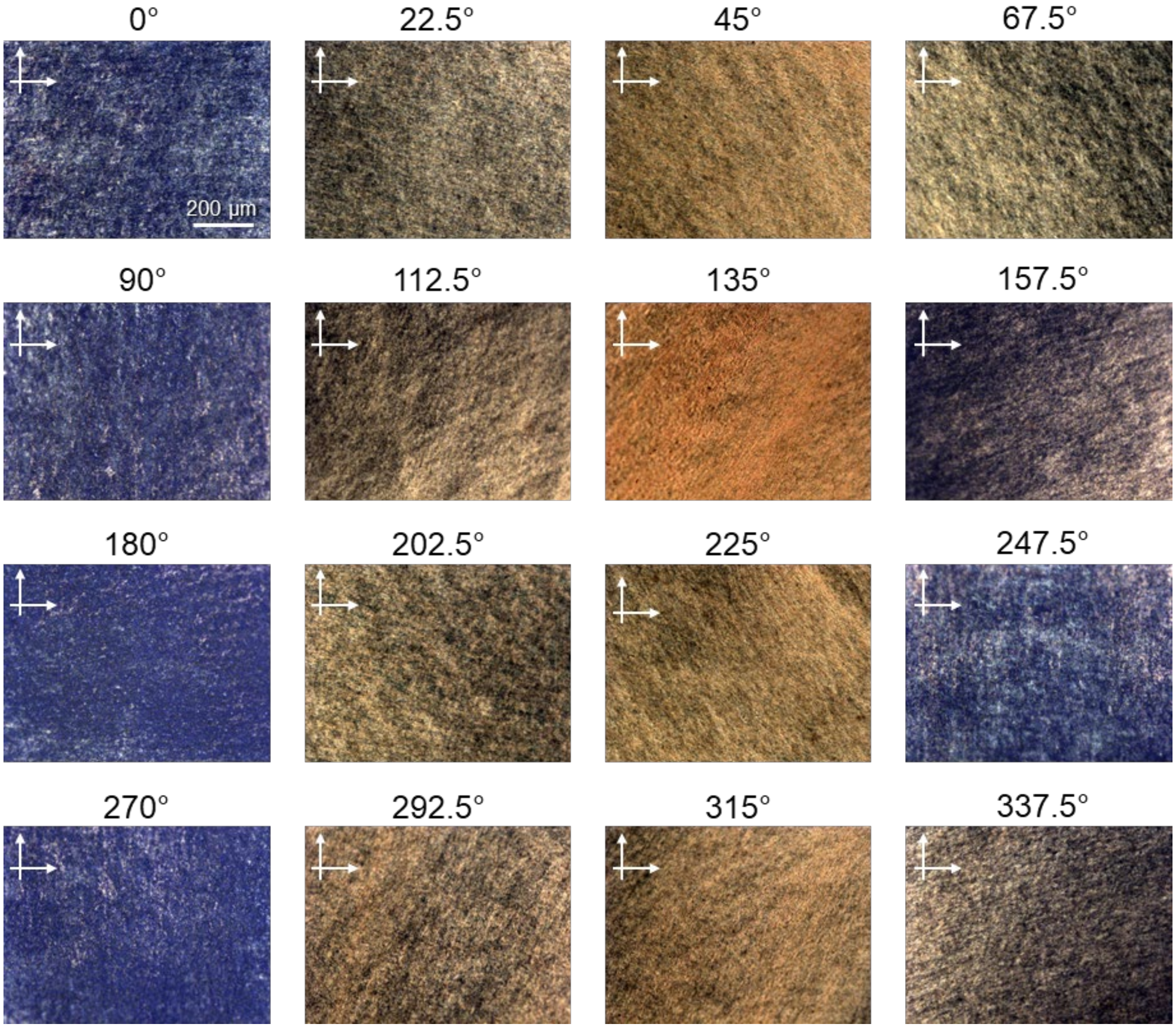

Figure S25. C-POM micrographs according to the position with angles of CNC/MNP composites film with 2.0 wt% of MNPs obtained under 400 rpm rotational magnetic field. Scale bars indicate 200 µm. All the images share the scale bar.

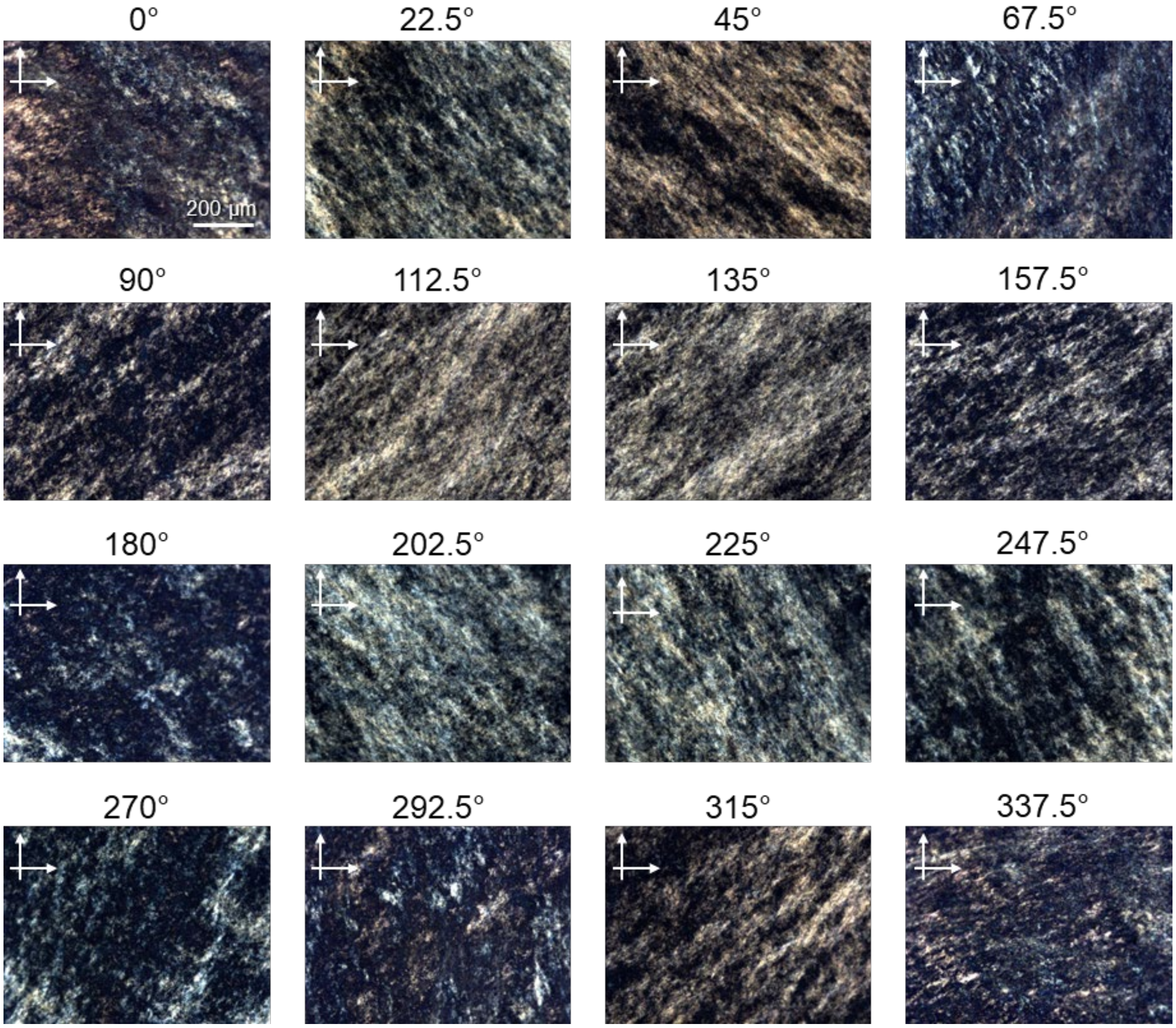

Figure S26. C-POM micrographs according to the position with angles of CNC/MNP composites film with 2.0 wt% of MNPs obtained under 800 rpm rotational magnetic field. All the images share the scale bar.

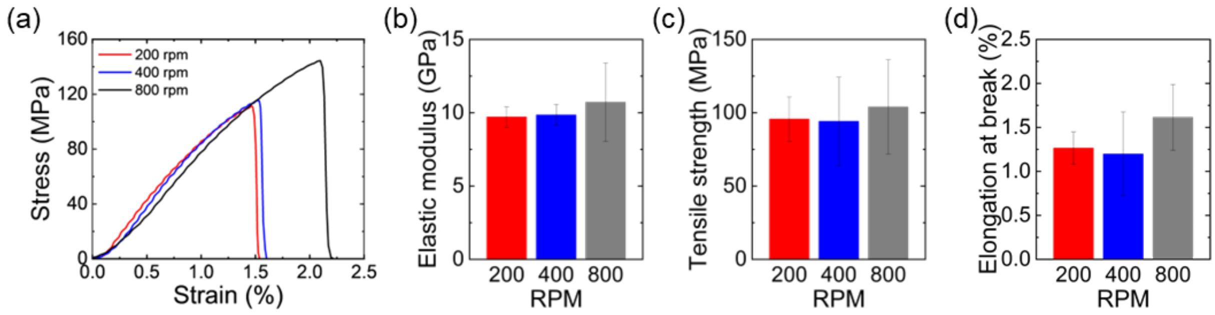

Figure S27. (a) Stress-strain curves, (b) elastic modulus, (c) tensile strength, and (d) elongation at break of the CNC/MNP composite film with different rotation rate.

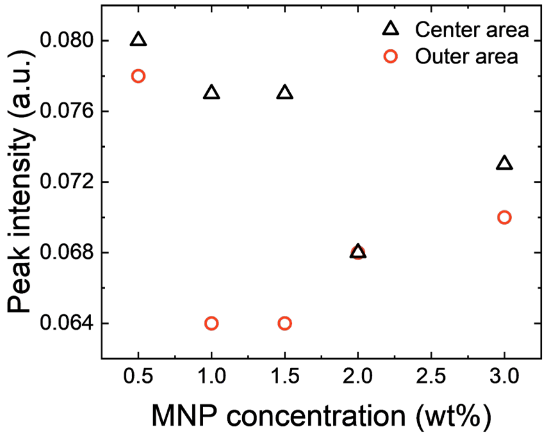

Figure S28. Peak height of CNC/MNP composite films obtained under rotational magnetic field with different concentrations.

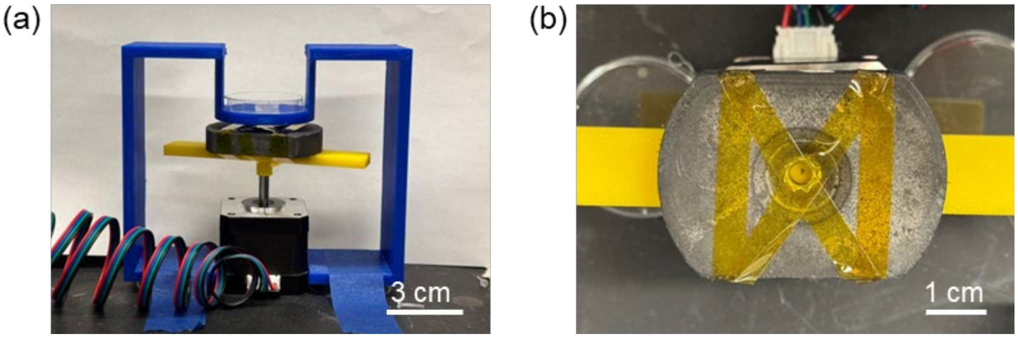

Figure S29. (**a**) Custom magnetic setup to regulate direction of rotation. (**b**) Shape of permanent magnet.

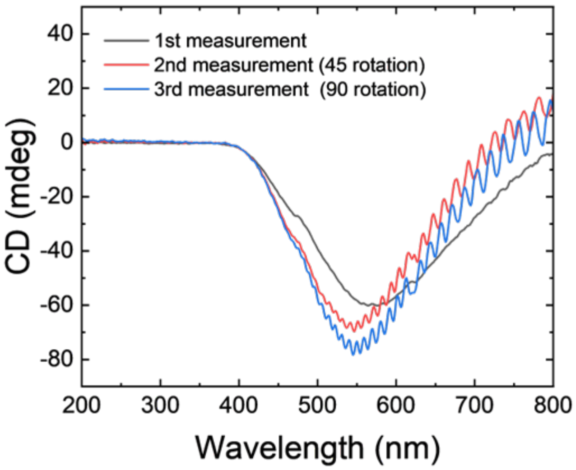

Figure S30. Multiple measurements of CD signals at the center of CNC/MNP composite films.

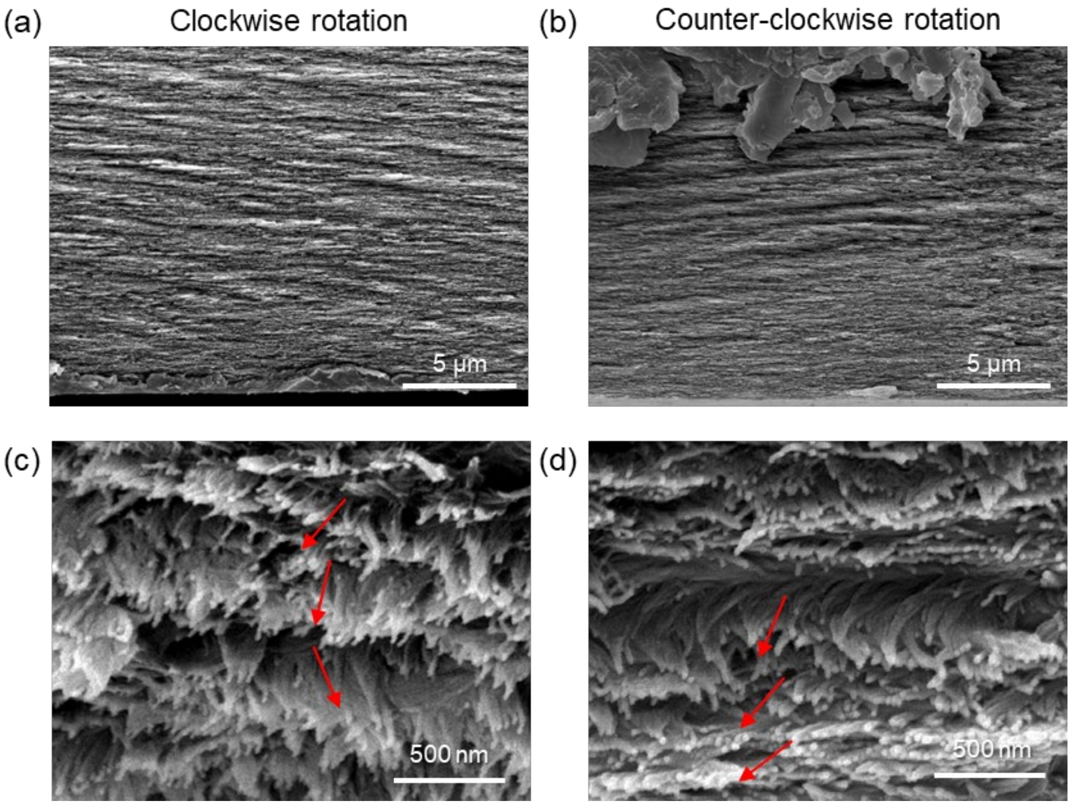

Figure S31. SEM micrographs of CNC/MNP composite films evaporated under (**a, c**) clockwise and (**b, d**) counter-clockwise rotational magnetic field. Red arrows indicate the direction of CNCs.
